# Time-Dependent Effects of Inotropic Exposure During Venoarterial Extracorporeal Membrane Oxygenation Support on the Probability of Extracorporeal Membrane Oxygenation Weaning Failure

**DOI:** 10.1097/CCM.0000000000007338

**Published:** 2026-09-25

**Authors:** Myrthe P.J. van Steenwijk, Lara C.A. Pladet, Atreyu van Esch, Hong Jun Shi, Dirk W. Donker, Yannick Taverne, Jeroen J.H. Bunge, Carlos V. Elzo Kraemer, Jacinta J. Maas, Leon J. Montenij, Luuk C. Otterspoor, Erik Scholten, Laurien van Koppenhagen, Joost van Rosmalen, Diederik Gommers, Alina A. Constantinescu, Rudolf A. de Boer, Jacob E. Møller, Dimitris Rizopoulos, Christiaan L. Meuwese

**Affiliations:** 1 Department of Intensive Care Adults, Erasmus Medical Center, Rotterdam, The Netherlands.; 2 Department of Cardiology, Thorax Center, Cardiovascular Institute, Erasmus Medical Center, Rotterdam, The Netherlands.; 3 Department of Intensive Care, University Medical Center Utrecht, Utrecht, The Netherlands.; 4 Department of Cardiovascular and Respiratory Physiology, University of Twente, Enschede, The Netherlands.; 5 Department of Cardiothoracic Surgery, Erasmus Medical Center, Rotterdam, The Netherlands.; 6 Department of Intensive Care, Leiden University Medical Center, Leiden, The Netherlands.; 7 Department of Intensive Care, Catharina Hospital Eindhoven, Eindhoven, The Netherlands.; 8 Department of Intensive Care, Sint Antonius Hospital, Nieuwegein, The Netherlands.; 9 Department of Intensive Care, Isala, Zwolle, The Netherlands.; 10 Department of Epidemiology & Health Economics, Julius Center for Health Sciences and Primary Care, University Medical Center Utrecht, Utrecht University, Utrecht, The Netherlands.; 11 Department of Cardiology, Copenhagen University Hospital–Rigshospitalet, Denmark.; 12 Departments of Biostatistics, Erasmus Medical Center, Rotterdam, The Netherlands.

**Keywords:** cardiogenic shock (CS), cumulative inotropic exposure, ECMO weaning failure, Inotropic Score (IS), joint model, venoarterial extracorporeal membrane oxygenation (VA ECMO)

## Abstract

**OBJECTIVES:**

During venoarterial extracorporeal membrane oxygenation (VA ECMO), organ perfusion no longer entirely depends on native cardiac output, which may reduce the therapeutic advantage of inotropic drugs relative to their risks. We hypothesized that, in this setting, higher cumulative inotropic exposure would be associated with impaired cardiac recovery.

**DESIGN:**

Observational cohort study on the effects of longitudinal inotropic exposure during VA ECMO support. Temporal changes in inotropic support during VA ECMO support were evaluated. Cumulative inotropic exposure per day during VA ECMO support was calculated using the area under the curve of a standardized inotropic score. These cumulative inotropic scores were associated with the risk of ECMO weaning failure up to 30 days (a composite of death, ongoing need for ECMO support, or the need for heart replacement therapy), mortality, and complications, using joint modeling techniques.

**SETTING:**

Six hospitals in the Netherlands.

**PATIENTS:**

Adult patients with severe cardiogenic shock (CS) receiving VA ECMO support between 2018 and 2024.

**INTERVENTIONS:**

None.

**MEASUREMENTS AND MAIN RESULTS:**

Out of 440 patients with various causes of CS, 235 patients (53.4%) developed ECMO weaning failure. Inotropic support remained largely unchanged in the days following VA ECMO initiation. Adjusted joint models showed that a higher cumulative inotropic exposure during VA ECMO support was associated with an increased risk for ECMO weaning failure (hazard ratio [HR], 1.04; 95% CI, 1.00–1.07), 30-day mortality (HR, 1.04; 95% CI, 1.01–1.08), arrhythmias (HR, 1.05; 95% CI, 1.01–1.08), and protracted lactate clearance (HR, 0.96; 95% CI, 0.92–0.99).

**CONCLUSIONS:**

In patients requiring VA ECMO support for refractory CS, inotropic support is often maintained at similar levels after ECMO initiation. Higher cumulative inotropic exposure was associated with an increased risk for ECMO weaning failure. Prospective studies are needed to investigate whether inotropes can be tapered or even stopped and whether such a strategy could improve cardiac recovery.

**KEY POINTS**

**Question:** How is inotropic medication currently used during venoarterial extracorporeal membrane oxygenation (VA ECMO) support, and how does cumulative inotropic exposure influence VA ECMO-related outcomes?

**Findings:** During VA ECMO support, dosages of inotropic drugs remained largely unchanged. A higher cumulative exposure to inotropic medications during VA ECMO support was associated with an increased risk for ECMO weaning failure, 30-day mortality, arrhythmias, and protracted clearance of lactate.

**Meaning:** These findings highlight the need for future interventional studies to evaluate the feasibility and safety of tapering or even stopping inotropic drugs in the setting of VA ECMO to improve cardiac recovery.

Cardiogenic shock (CS) is an increasingly occurring, life-threatening condition (1, 2), in which short-term prognosis mainly depends on the extent of evolving organ failure. Initial management therefore centers around immediate restoration of systemic blood flow, typically through the use of inotropes (3–7).

Temporary mechanical circulatory support (tMCS) systems, like venoarterial extracorporeal membrane oxygenation (VA ECMO), can safeguard organ perfusion and thereby uncouple organs’ perfusion dependency on native cardiac output, rendering the initial indication for inotropic therapy less relevant. This setting could provide a safety window during which inotropic medication could be reduced and possibly even stopped. However, little is known about the risk-benefit ratios and optimal use of inotropes in the setting of VA ECMO. On one hand, inotropic support may enhance cardiac contractility and unload the left ventricle (LV) (8), thereby preventing pulmonary edema or intracavitary thrombus formation. On the other hand, inotropes may further injure an already failing myocardium through their cardiotoxic effects (4, 9, 10). The uncertainty surrounding the risk-benefit ratio of inotropes in the setting of VA ECMO is reflected in the lack of clear management protocols for their use.

We hypothesized that inotropic drugs are often continued during VA ECMO support in their original dosages and that a higher cumulative inotropic exposure may be associated with reduced cardiac recovery. Therefore, we studied temporal changes in inotropic dosages during VA ECMO support and associated the cumulative inotropic exposure with the probability of ECMO weaning failure in patients requiring VA ECMO support for severe CS.

## MATERIALS AND METHODS

### Study Design and Setting

We used data from the PREdiCtion of weanability, Survival, and functional outcomEs after extracorporeal life support (PRECISE ECLS) cohort (National Clinical Trial [NCT] number: NCT05444764). This multicenter cohort study included all patients who received ECMO for refractory respiratory and/or cardiocirculatory failure between 2016 and 2024 in ICUs of six participating Dutch hospitals. The study was approved by the Institutional Review Board of the University Medical Center of Utrecht and, thereafter, by local ethical committees of participating centers (**Supplement 1**, https://links.lww.com/CCM/I59). Informed consent was obtained in accordance with the requirements of each local ethics committee. For the current study, we selected all individuals of 18 years or older receiving VA ECMO support for severe CS. Patients were excluded if they had received VA ECMO support for less than 1 day, as deaths within this period are generally attributable to causes other than prolonged cardiac recovery (11). Also, we excluded patients who underwent a second ECMO run or had a left ventricular assist device (LVAD) implanted before VA ECMO initiation. This study adhered to the principles of the Declaration of Helsinki (75th World Medical Association General Assembly, Helsinki, Finland, October 2024) and Good Clinical Practice.

### Data Collection

Patient data were collected from their medical records and included general characteristics (*e.g., age, sex, comorbidities, Acute Physiology and Chronic Health Evaluation IV [APACHE IV] score, and hospital and ICU admission dates*), detailed VA ECMO-related characteristics (*e.g., indication, sizes, and sites of cannulas*), and data on hemodynamics, adjuvant therapies on ECMO (*e.g., mechanical unloading strategies*), and the occurrence of ECMO-related complications. Prospectively defined definitions of relevant variables are provided in **Supplement 2** (https://links.lww.com/CCM/I59). Patient data were collected on a daily basis at 6 am from day 1 to 7 and then weekly on days 14, 21, and 28, as long as patients were receiving ECMO support. Inotropic exposure during VA ECMO support was calculated daily during the first 7 days by summarizing all inotropic agents and their dosages using a validated and standardized Inotropic Score (IS) (12):


$$\begin{array}{cc} \; & 100*Epinephrine\left(\mu g/kg/min\right)\\ \; & +\;50*levosimendan\left(\mu g/kg/min\right)\\ \; & +\;10*milrinone\;\left(\mu g/kg/min\right)\\ \; & +\;dobutamine\left(\mu g/kg/min\right)\\ \; & +\;dopamine\left(\mu g/kg/min\right)+enoximone\left(\mu g/kg/min\right). \end{array}$$

An increment of one unit in IS corresponds to an increase of 1 µg/kg/min dobutamine or a 0.1 µg/kg/min increase in milrinone. We deliberately left out norepinephrine from the equation because it is predominantly recommended for vasoplegic shock (13), and carries a disproportionately large weight in the composite score calculation, which would cause the score to predominantly reflect vasopressor exposure rather than true inotropic support.

Before initiation of VA ECMO, inotropes were titrated according to guideline recommendations to achieve a cardiac index greater than 1.8 L/min/m^2^ (preferably > 2.2 L/min/m^2^), a mean arterial pressure (MAP) greater than or equal to 65 mm Hg, a lactate level less than 2.0 mmol/L, and a venous oxygen saturation greater than or equal to 65% (14).

### Outcomes

The primary endpoint of the study was ECMO weaning failure until 30 days, used as a proxy for insufficient cardiac recovery, and defined as a composite of death, ongoing or renewed need for ECMO support, or the need for heart transplantation or LVAD implantation. Other outcomes included 30-day and 1-year mortality, ECMO support duration, ICU and hospital admission duration, the need for mechanical LV unloading devices, occurrence of arrhythmias, initiation of renal replacement therapy (RRT), occurrence of intracardiac or aortic root thrombosis, and time to lactate clearance (Supplement 2, https://links.lww.com/CCM/I59).

### Statistical Analysis

Data were expressed as numbers and percentages (%), medians and interquartile ranges (IQRs), or as mean ± sd, as appropriate. To compare patients with different inotropic exposure, patients were categorized into three equal groups according to tertiles of cumulative inotropic exposure. Cumulative inotropic exposure was calculated by the area under the curve (AUC) of the IS trajectory during VA ECMO support, divided by the number of ECMO support days with a maximum of 7 days (**Supplement 3**, https://links.lww.com/CCM/I59). Baseline characteristics and unadjusted outcomes were compared across these tertile groups by using an analysis of variance (normally distributed data) or Kruskal-Wallis (nonnormally distributed data) test for continuous variables and the chi-square test or Fisher exact test for categorical variables. ECMO support duration and ICU and hospital length of stay were compared between 30-day survivors to minimize bias due to differences in early mortality. A Kaplan-Meier curve with log-rank test was constructed to illustrate the cumulative incidence of ECMO weaning failure per group over the first 30 days following VA ECMO initiation.

To visualize changes in ISs over time, we used an Alluvial plot stratified by tertiles of baseline (day 1) IS, illustrating within-group trajectories and ECMO weaning outcomes over time. To identify whether certain subgroups were more likely to receive inotropes during VA ECMO support, we plotted mean IS trajectories in subgroups based on central venous pressure (CVP), Fio_2_ requirement, baseline pulse pressure (difference between systolic and diastolic blood pressure), and the use of mechanical unloading devices. In addition, temporal trajectories of hemodynamic parameters (e.g., heart rate, MAP, fluid balance, ECMO blood flow, and sweep gas flow) were assessed during the first 5 days of VA ECMO support and compared between the three cumulative inotropic exposure groups using Locally Estimated Scatterplot Smoothing-generated lines with 95% CIs.

The association between time-dependent inotropic exposure and patient outcomes was studied through joint models, which combine linear mixed models (LMM) with a Cox model to study associations between time-varying covariates and time-to-event outcomes (15). The longitudinal trajectory of the IS during the first 7 days of VA ECMO support was included in a Cox proportional hazards model to assess the association between IS trajectory and time-to-ECMO weaning failure. Apart from baseline confounders, additional LMMs were incorporated into the joint model to adjust for important time-varying confounders. For each of these time-varying confounders, the value from the preceding day was used to prevent the risk of reversed causality (16). We incorporated the *AUC* of the IS trajectory as a functional form into the Cox submodel as we hypothesized that the detrimental effects of inotropic exposure would be cumulative (17, 18). To compare patients with different VA ECMO support durations, the AUC was normalized by the number of VA ECMO support days with a maximum of 7 days. Time-to-event secondary outcomes were also analyzed using separate joint models. Details about the development of the model, including confounder selection, are provided in **Supplements 4** and **5** (https://links.lww.com/CCM/I59).

As a sensitivity analysis, we refitted joint models after excluding patients with a mechanical unloading device at baseline or a baseline pulse pressure less than 15 mm Hg, as these situations likely reflect a more critically ill subpopulation in whom inotropes may have been indicated to maintain adequate transpulmonary blood flow (19, 20). In addition, we performed a competing risk analysis to account for patients in whom VA ECMO was discontinued after irreversible neurologic injury, as this precludes the possibility of ECMO weaning failure due to poor cardiac recovery. In another sensitivity analysis, we included norepinephrine dosage in the calculation of the IS (21), as norepinephrine also exerts some inotropic effects (22). Finally, to dissect whether adverse effects of inotropes on ECMO weaning would be preferentially attributable to phosphodiesterase inhibitors (PDEIs; milrinone, enoximone) or beta-receptor agonists (BRA; dobutamine, dopamine, epinephrine), we studied the association between the ratio of these drugs (PDEI/BRA) and ECMO weaning failure, adjusting for baseline total IS to assess the effect of the relative proportion between the two inotropic drug types rather than the absolute dose.

The proportion of missing values was minimal, with only one model covariate containing missing values (**Supplement 6**, https://links.lww.com/CCM/I59). Missing data for the vasopressor score before VA ECMO initiation (*n* = 19; 4.3%) were imputed using multiple imputation with chained equations. *p* value of less than or equal to 0.05 were considered statistically significant. Statistical analyses were performed in R (Version 4.4.2, 2024.09.01, Vienna, Austria) using the packages mice (v3.16.0) for multiple imputation, survival (v3.5.7) for time-to-event analyses, and JMbayes2 (v0.5.0) for joint modeling.

## RESULTS

Out of 808 patients who received ECMO support between 2016 and 2024, 440 patients fulfilled the inclusion criteria (**Supplement 7**, https://links.lww.com/CCM/I59). Median age at VA ECMO initiation was 58 years (IQR, 48–68 yr), 70.2% of patients were male, and the majority received VA ECMO after cardiac surgery (37.3%), followed by extracorporeal cardiopulmonary resuscitation (ECPR; 25.7%). Across the three IS tertiles based on the AUC normalized per day (low, < 1.5; moderate, 1.5–3.0; high, >3.0), patients in the lowest tertile were least likely to have an unloading device at the time of VA ECMO initiation. 76.8% of patients received any inotropic agent during VA ECMO support, with patients in the highest IS tertile most often receiving epinephrine and dobutamine, whereas milrinone and enoximone were most frequently administered in the moderate tertile group (**Supplement 8**, https://links.lww.com/CCM/I59). No statistically significant differences were observed in other baseline characteristics (**Table 1**; Supplement 8, https://links.lww.com/CCM/I59). Before VA ECMO initiation, patients with higher inotropic exposure had lower cardiac output, MAP, and venous oxygen saturation, along with higher lactate levels (**Supplement 9**, https://links.lww.com/CCM/I59). However, no association was observed between inotropic exposure and the presence of pulmonary edema on pre-ECMO chest radiography (Table 1).

**TABLE 1.** Baseline Characteristics Across Tertiles of Inotrope Exposure at Venoarterial Extracorporeal Membrane Oxygenation Initiation

Characteristic	Total	Lowest tertile (IS AUC < 1.50)	Moderate tertile (IS AUC 1.50–3.00)	Highest tertile (IS AUC > 3.00)	*p* ^d^
Number of patients	*n* = 440	*n* = 147	*n* = 147	*n* = 146	
Age, median (IQR)	58.0 (48.0–68.0)	58.0 (47.8–67.3)	57.0 (49.0–65.0)	60.0 (48.0–70.5)	0.449
Sex, male, *n* (%)	309 (70.2)	103 (70.1)	102 (69.4)	104 (71.2)	0.929
Body mass index, median (IQR)	26.6 (24.1–30.1)	26.3 (23.9–30.0)	27.3 (24.7–31.1)	26.5 (24.1–29.4)	0.107
Medical history, *n* (%)^a^					
Hypertension	138 (31.4)	40 (27.2)	52 (35.4)	46 (31.5)	0.551
Diabetes mellitus	71 (16.1)	19 (12.9)	27 (18.4)	25 (17.1)	0.573
Myocardial infarction	71 (16.1)	18 (12.2)	18 (12.2)	35 (24.0)	0.022
Coronary artery disease	83 (18.9)	21 (14.4)	23 (15.6)	39 (26.7)	0.030
Heart failure	97 (22.0)	33 (22.4)	29 (19.7)	35 (24.0)	0.592
Coronary artery bypass grafting	20 (4.5)	5 (3.4)	3 (2.0)	12 (8.2)	0.088
Percutaneous coronary intervention	63 (14.3)	20 (13.5)	17 (11.6)	27 (18.5)	0.281
Peripheral arterial disease	20 (4.5)	5 (3.4)	5 (3.4)	10 (6.8)	0.451
Chronic kidney disease	31 (7.0)	9 (6.1)	12 (8.2)	10 (6.8)	0.636
Charlson Comorbidity Index, median (IQR)	2.0 (2.0–4.0)	2.0 (2.0–4.0)	2.0 (2.0–4.0)	2.00 (2.0–4.0)	0.841
Acute Physiology and Chronic Health Evaluation IV score, mean (sd)	86.1 (33.9)	83.0 (32.2)	88.1 (33.0)	87.9 (36.9)	0.349
Pulmonary edema on X-ray pre-ECMO,^c^ *n* (%)	86 (27.8)	36 (34.6)	20 (20.4)	30 (33.7)	0.051
Vasopressor score, median (IQR)	25.0 (2.0–54.4)	25.6 (3.0–49.4)	20.9 (0.0–52.1)	33.0 (4.0–69.5)	0.110
ECMO indication, *n* (%)					0.021
Cardiomyopathy-CS	47 (10.7)	12 (8.2)	21 (14.3)	14 (9.6)	
Extracorporeal cardiopulmonary resuscitation	113 (25.7)	44 (29.9)	36 (24.5)	33 (22.6)	
Myocardial Infarction-CS	63 (14.3)	20 (13.6)	23 (15.6)	20 (13.7)	
Postcardiotomy-CS	164 (37.3)	45 (30.6)	50 (34.0)	69 (47.3)	
Other^b^	53 (12.0)	26 (17.7)	17 (11.6)	10 (6.8)	
Unloading device, *n* (%)					0.001
Intra-aortic balloon pump	124 (28.2)	28 (19.0)	40 (27.2)	56 (38.4)	
Impella	38 (8.6)	11 (7.5)	20 (13.6)	7 (4.8)	
None	276 (62.7)	106 (72.1)	87 (59.2)	83 (56.8)	
Other	2 (0.5)	2 (1.4)	0 (0.0)	0 (0.0)	

AUC = area under the curve, CS = cardiogenic shock, ECMO = extracorporeal membrane oxygenation, IQR = interquartile range, IS = inotropic score.

a All medical history variables reflect comorbidities or events occurring before the current hospital admission and do not include conditions serving as the indication for the present ECMO run.

b Myocarditis, congenital heart disease, intoxication, other.

c Available in 309 patients.

d Mean differences were tested using analysis of variance, median differences using the Kruskal-Wallis method, and categorical variables were compared using a χ^2^ test or Fisher exact test.

The IS at baseline (day 1) ranged from 0 to 40.6 (median, 2.5; IQR, 1.0–5.0). Categorization into low, moderate, and high baseline IS groups based on tertiles yielded the following cutoffs: less than 1.9, 1.9–4.0, and greater than 4.0, respectively (**Fig. 1**). A total of 72.6% of patients in the moderate or high IS tertiles at baseline received comparably high inotropic dosages before VA ECMO support. During VA ECMO support, over 80% of patients starting in the middle and highest tertiles either remained in the same IS tertile as baseline or died.

**Figure 1.**
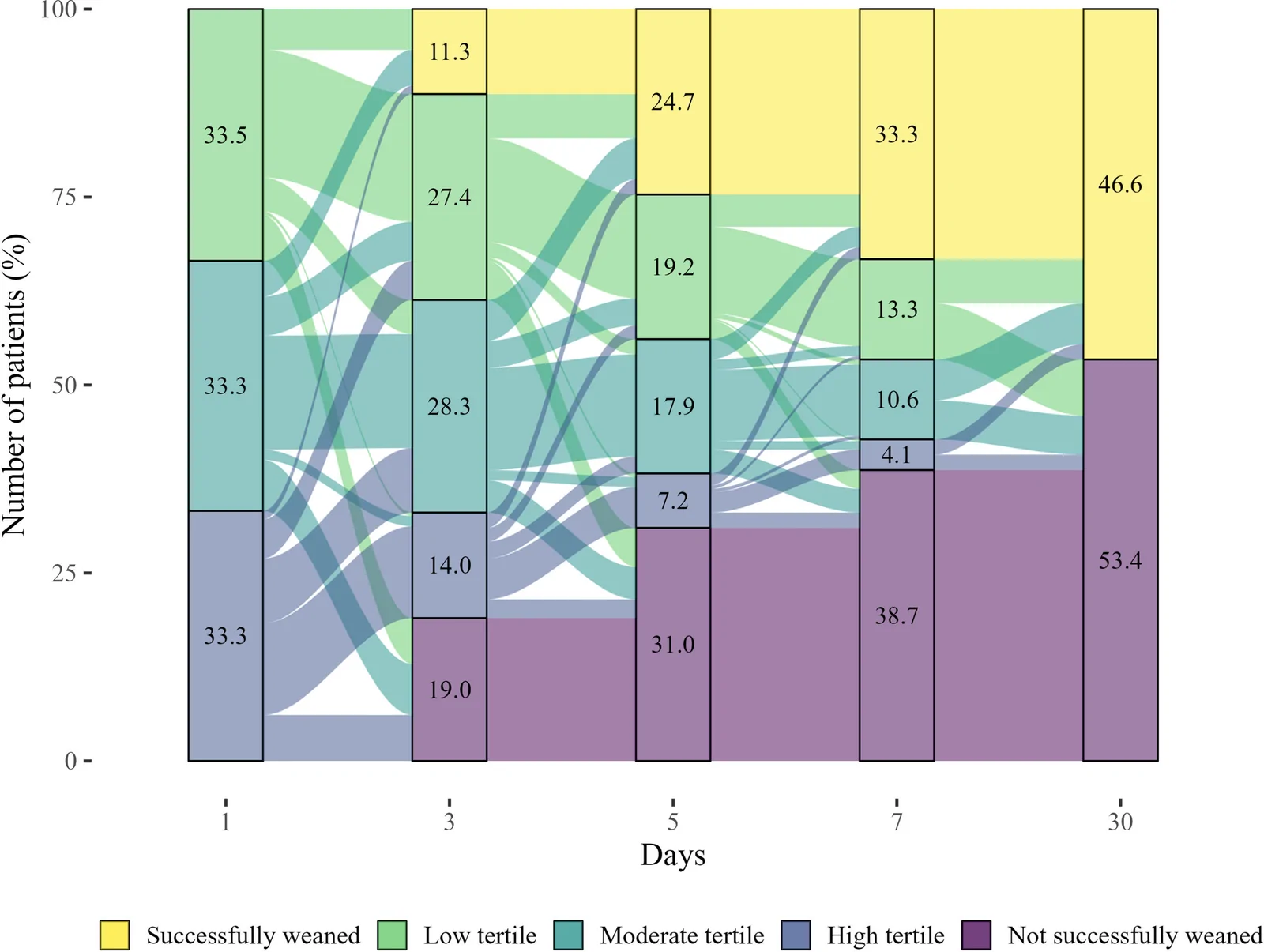
Alluvial plot showing the temporal changes across inotropic score categories. Inotropic score tertiles are based on the inotropic score baseline distribution (day 1). The plot illustrates changes in inotropic score tertiles from day 1 through days 3, 5, and 7 of extracorporeal membrane oxygenation (ECMO) support, and shows the eventual outcome of either successful or failed ECMO weaning over time.

The IS trajectory during VA ECMO support was comparable in subgroups stratified by pulse pressure (≤ 15 vs. > 15 mm Hg), CVP (≤ 10 vs. > 10 mm Hg), Fio_2_ requirement (≤ 40% vs. > 40%), and the presence of a mechanical unloading device (**Fig. 2**). Patients in the higher IS exposure group exhibited greater illness severity at baseline, reflected by higher heart rate, lactate levels, vasopressor scores, and Sequential Organ Failure Assessment scores, as well as increased support requirements, including higher VA ECMO blood flow and a more positive fluid balance. No clear difference was seen in Fio_2_ requirement. During ECMO support, lactate levels and fluid balance converged between groups, while other clinical parameters showed similar trends over time (**Supplements** 5 and **10**, https://links.lww.com/CCM/I59).

**Figure 2.**
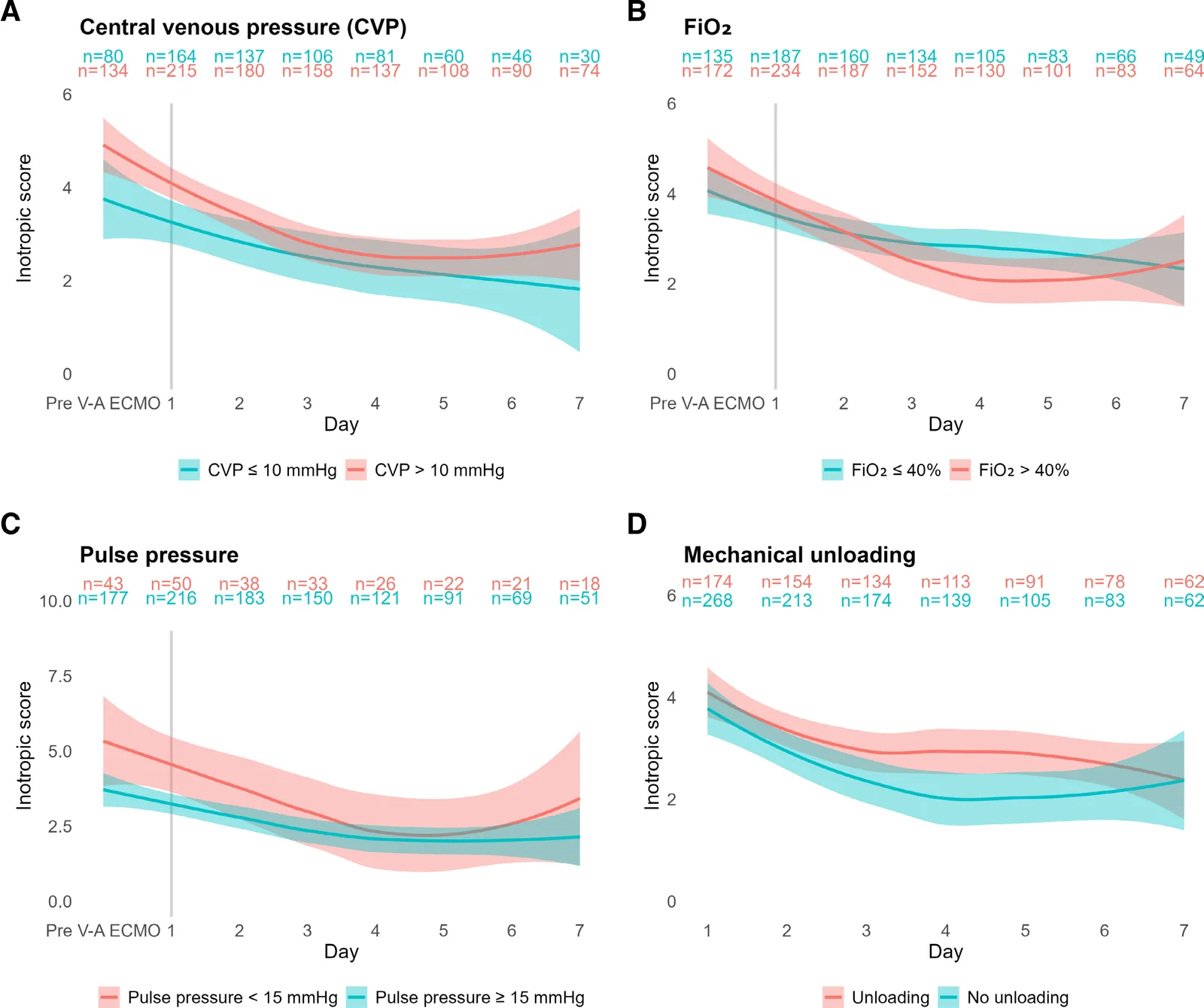
Mean Inotropic Score (IS) trajectories stratified by baseline values of pulse pressure, central venous pressure (CVP), Fio_2_ requirement, and mechanical unloading status. This figure depicts the mean inotropic score with corresponding 95% CIs during venoarterial extracorporeal membrane oxygenation (VA ECMO) support over a 7-d period. Patients are stratified into two groups based on **A**, baseline CVP using a cutoff of 10 mm Hg; **B**, baseline Fio_2_ requirement using a cutoff of 40% (the median); **C**, baseline pulse pressure (systolic minus diastolic blood pressure) using a cut-off of 15 mm Hg; and **D**, the presence of a mechanical unloading device at the initiation of VA ECMO support. Before VA ECMO initiation, patients with lower pulse pressure, higher CVP, and higher Fio_2_ requirements tended to have higher inotropic scores. During VA ECMO support, trajectories of these parameters showed no clear differences. Patients with a mechanical unloading device at baseline exhibited slightly higher inotropic scores throughout ECMO support. Patient numbers contributing to each time point are shown.

Out of 440 patients, 235 patients (53.4%) could not be successfully weaned from ECMO support at 30 days after VA ECMO initiation due to the need for persistent ECMO support (*n* = 2; 0.9%), direct LVAD implantation (*n* = 23; 9.8%), heart transplantation (*n* = 3; 1.3%), or death (*n* = 207; 88.0%). One year after VA ECMO initiation, mortality was 57.8% (*n* = 245). The incidence of ECMO weaning failure was highest in the high cumulative IS tertile (61.0%) vs. the groups receiving moderate (52.4%) and low cumulative dosages (46.9%; **Table 2**; **Fig. 3**). Survivors at 30 days after ECMO initiation in the highest cumulative IS tertile had significantly longer ICU lengths-of-stay (25.0 d [IQR, 11.5–35.0 d]) as compared with patients in the low and moderate cumulative tertiles (17.0 d [IQR, 7.0–36.0 d] and 14.0 d [IQR, 8.0–32.5 d], respectively; Table 2). No significant differences were observed in other secondary outcomes (Table 2).

**TABLE 2.** Primary and Secondary Outcomes, Unadjusted for Confounders Stratified for Inotrope Exposure

Outcomes	Total	Lowest tertile (IS AUC < 1.50)	Moderate tertile (IS AUC 1.50–3.00)	Highest tertile (IS AUC > 3.00)	*p*
	440	147	146	147	
Weaning failure, *n* (%)	235 (53.4)	69 (46.9)	77 (52.4)	89 (61.0)	0.041
Heart transplantation, *n* (%)	3 (0.7)	0 (0.0)	2 (1.4)	1 (0.7)	0.363
Left ventricular assist device, *n* (%)	23 (5.2)	7 (4.8)	9 (6.1)	7 (4.8)	0.828
Mortality at 30 d, *n* (%)	207 (47.0)	60 (40.8)	68 (46.3)	79 (54.1)	0.056
Mortality at 1 yr, *n* (%)	245 (57.8)	80 (57.1)	76 (53.5)	89 (62.7)	0.270
Extracorporeal membrane oxygenation days,^a^ median (IQR)	4.0 (3.0–7.0)	4.0 (2.0–7.0)	4.0 (2.0–7.0)	5.0 (3.0–9.0)	0.053
ICU LoS,^a^ median (IQR)	19.0 (9.0–34.0)	17.0 (7.0–36.0)	14.0 (8.0–32.5)	25.0 (11.5–35.0)	0.050
Hospital LoS,^a^ median (IQR)	37.5 (18.0–61.3)	33.0 (16.0–62.0)	34.0 (15.5–57.5)	43.0 (24.5–62.5)	0.131
Intracardiac or aortic root thrombosis	11 (2.5)	1 (0.7)	3 (2.0)	7 (4.8)	0.072
Additional need for adjunct mechanical left ventricle unloading^b^	23 (8.3)	8 (7.5)	7 (8.3)	7 (8.0)	0.979
Renal replacement therapy	143 (32.5)	44 (29.9)	49 (33.3)	50 (34.2)	0.781

AUC = area under the curve, IQR = interquartile range, IS = inotropic score, LoS = length of stay.

a Measured in patients who survived until 30 d.

b Measured in a subset of patients in whom an unloading device was not present at baseline, *n* = 276.

Cumulative inotropic exposure groups are based on tertiles of the area under the curve of the inotropic score trajectory normalized by the number of extracorporeal membrane oxygenation support days, with a maximum of 7 d.

**Figure 3.**
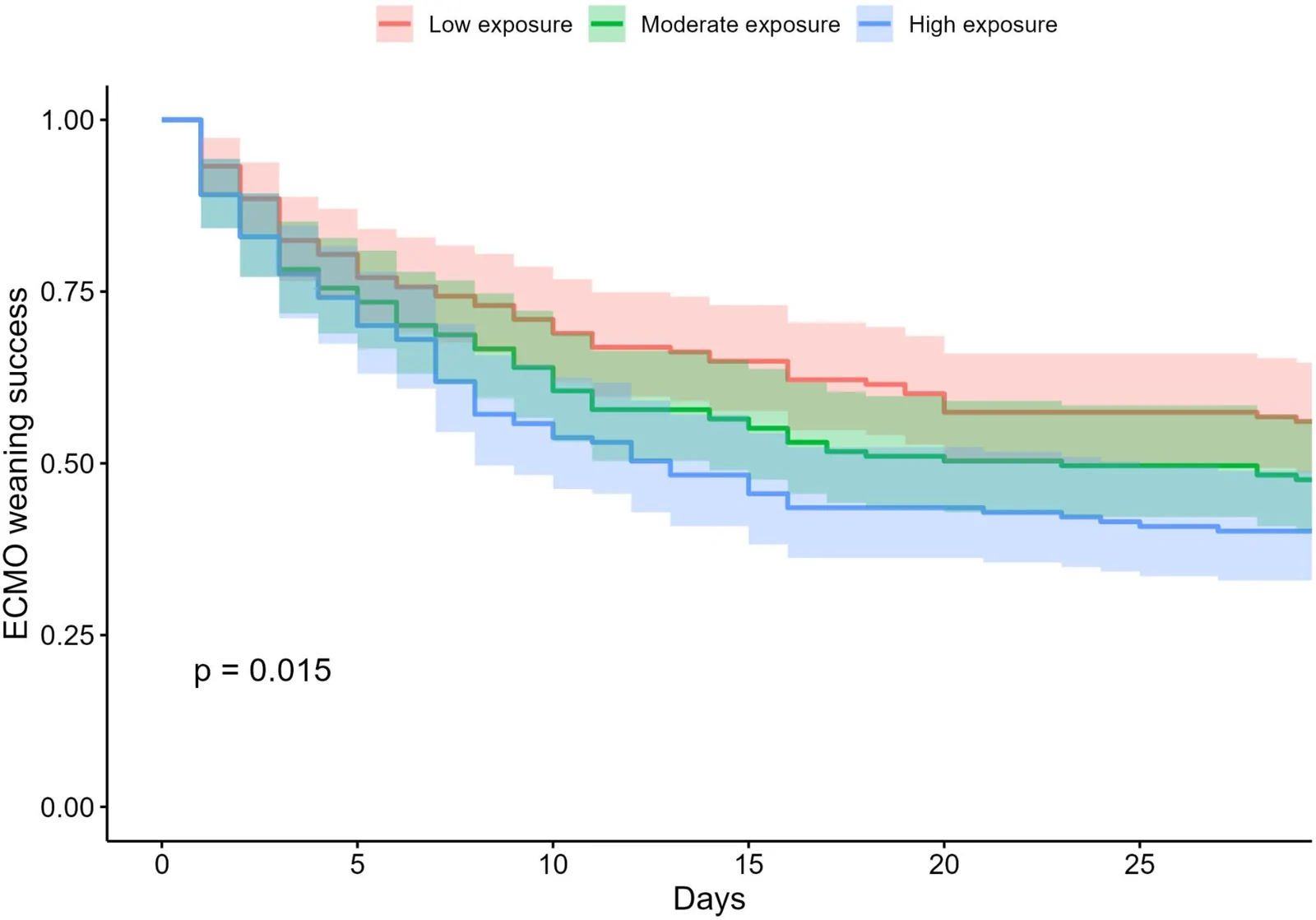
The occurrence of extracorporeal membrane oxygenation (ECMO) weaning failure at 30 d after support initiation. Kaplan-Meier analysis of the occurrence of ECMO weaning failure over the 30 d after ECMO initiation, where groups are based on the area under the curve of the inotropic score trajectory during ECMO support, normalized per day. Subsequently, these scores were divided into three groups based on tertiles.

In joint models adjusting for baseline and time-varying covariates (**Supplement 11**, https://links.lww.com/CCM/I59), a one-unit increase in cumulative IS was associated with a statistically significant four percent higher risk for ECMO weaning failure (hazard ratio [HR], 1.04; 95% CI, 1.00–1.07; **Table 3**). This additional one-unit increase in cumulative IS corresponds to, for example, a sustained 1 µg/kg/min increase in dobutamine dosage during VA ECMO support. Accordingly, two- or three-unit higher cumulative ISs were associated with an increased hazard of approximately 8% (1.04^2^) and 12% (1.04^3^), respectively. An increase in cumulative IS was also significantly associated with a higher risk for 30-day mortality (HR, 1.04; 95% CI, 1.01–1.08), arrhythmias (HR, 1.05; 95% CI, 1.02–1.09), and protracted lactate clearance (< 2 mmol/L) during VA ECMO support (HR, 0.96; 95% CI, 0.92–0.99; *n* = 318; Table 3). Cumulative IS was not associated with the probability of RRT initiation.

**TABLE 3.** Outcomes of Joint Models Assessing the Association Between Cumulative Inotrope Exposure During Venoarterial Extracorporeal Membrane Oxygenation Support and Patient Outcomes

Outcomes	Unadjusted Joint Models			Adjusted Joint Models		
	Hazard Ratio	95% CI	*p*	Hazard Ratio	95% CI	*p*
Primary outcome						
ECMO weaning failure^a^	1.04	0.99–1.10	0.124	1.04^f^	1.00–1.07	0.038
Mechanically unloaded patients excluded^b^	1.02	0.99–1.03	0.145	1.10^f^	1.04–1.16	< 0.001
Baseline pulse pressure < 15 mm Hg excluded^c^	1.07	1.00–1.13	0.058	1.05^f^	1.01–1.10	0.027
Secondary outcomes						
Mortality^a^	1.05	1.01–1.09	0.027	1.04^f^	1.01–1.08	0.019
Arrhythmias^e^	1.05	1.00–1.10	0.057	1.05^g^	1.01–1.08	0.007
Lactate clearance^d,e^	0.96	0.92–0.99	0.206	0.96^h^	0.92–0.99	0.035
Renal replacement therapy^e^	1.00	0.91–1.09	0.976	0.99^h^	0.93–1.04	0.634

APACHE IV = acute physiology and chronic health evaluation, BMI = body mass index, VA ECMO = venoarterial extracorporeal membrane oxygenation.

a Measured until 30 d after VA ECMO initiation.

b Determined in *n* = 268 patients.

c Determined in *n* = 357 patients.

d Determined in *n* = 318 patients. Lactate clearance is defined as the time until reaching a lactate < 2 mmol/L.

e Measured during and until 24 hr after ECMO support.

f Adjusted for confounders at baseline (age, BMI, sex, Charlson Comorbidity Index, APACHE IV score, vasopressor score, ECMO indication, hospital, mechanical unloading device) and longitudinal variables during ECMO support (heart rate, the occurrence of left ventricular overload and arrhythmias).

g Adjusted for confounders at baseline (age, BMI, sex, Charlson Comorbidity Index, APACHE IV score, ECMO indication, mechanical unloading device) and longitudinal variables during ECMO support (the occurrence of left ventricular overload).

h Adjusted for confounders at baseline (age, BMI, sex, Charlson Comorbidity Index, APACHE IV score, vasopressor score, ECMO indication, mechanical unloading device) and longitudinal variables during ECMO support (the occurrence of left ventricular overload).

Subgroup analyses of postcardiotomy patients and non-ECPR patients did not significantly alter our findings (**Supplement 12**, https://links.lww.com/CCM/I59). After excluding patients with a pulse pressure lower than 15 mm Hg or a mechanical unloading device at baseline, cumulative inotropic exposure remained associated with a higher risk for ECMO weaning failure (HR, 1.05; 95% CI, 1.01–1.10; *n* = 357 and HR, 1.10; 95% CI, 1.04–1.16; *n* = 268, respectively; Table 3). Hazards for ECMO weaning failure were not significantly associated with the ratio of BRA/PDEI agents (HR, 0.97; 95% CI, 0.91–1.02). Including norepinephrine dosage in the calculation of IS did not materially alter our findings (HR, 1.02; 95% CI, 1.01–1.02). In the competing risk analysis, cumulative IS was significantly associated with death due to nonneurologic mortality (HR, 1.04; 95% CI, 1.00–1.09) but not with death due to neurologic causes (HR, 1.02; 95% CI, 0.96–1.06; **Supplement 13**, https://links.lww.com/CCM/I59).

## DISCUSSION

In our study, we found that dosages of inotropic drugs remained largely unchanged during VA ECMO support and that a higher cumulative exposure to inotropic drugs was associated with an increased risk for ECMO weaning failure, 30-day mortality, arrhythmias, and protracted lactate clearance. Although limited by its observational study design, these findings support the hypothesis that, in the context of VA ECMO support, the risks associated with inotrope use may outweigh their potential benefits.

Our observation that inotropic drug dosages remained largely unchanged during VA ECMO support aligns with results from the extracorporeal life support in infarct-related cardiogenic shock (ECLS-SHOCK) trial in which a higher number of patients in the VA ECMO arm received inotropes for a longer period of time as compared with the control group (23). This higher need for inotropes is traditionally explained by the occurrence of “LV overload” secondary to ventriculoaortic uncoupling caused by aortic pressurization due to (retrograde) extracorporeal blood flow (24). This apparent reasoning, however, does not seem to hold: first, a recent report challenged the concept of VA ECMO-induced LV overload, showing that pulmonary capillary wedge pressures slightly decreased, rather than increased, with higher VA ECMO blood flow (25). Second, in our data, changes in dosages of inotropic drugs were not associated with the presence of pulmonary edema before ECMO initiation, the need for mechanical LV unloading during VA ECMO support, or with trends in pulse pressure, Fio_2_, or CVP, representing proxies for LV overload. In addition, excluding patients who received mechanical LV unloading strengthened the observed association, indicating that the relationship between inotropic exposure and weaning failure is not solely driven by unloading-related disease severity. These findings suggest that inotrope management may be partly subjective and arbitrary, reflecting limited evidence to guide their use in this setting.

Our second important finding was that a higher cumulative exposure to inotropes during VA ECMO support was associated with an increased risk for ECMO weaning failure and other adverse events. These findings are supported by previous studies which have consistently failed to demonstrate a survival benefit for the use of inotropes in patients with varying forms of heart failure or CS, while instead reporting associations with increased risk of arrhythmias, refractory shock, myocardial ischemia, and mortality (7, 10, 26). One of the largest observational studies to date, including more than 16.000 CS patients, demonstrated that inotropes were associated with a significant 1.8-fold higher risk of mortality (27). In addition, the OptimaCC trial showed that epinephrine vs. norepinephrine was associated with a markedly higher risk of refractory CS leading to premature trial termination (28). Likewise, the Outcomes of a Prospective Trial of Intravenous Milrinone for Exacerbations of Chronic Heart Failure trial demonstrated that the use of milrinone, compared with placebo, in patients with ischemic cardiomyopathy was associated with prolonged or recurrent hospitalization and a trend toward increased mortality (5). Recently, the Levosimendan to Facilitate Weaning From ECMO in Patients With Severe Cardiogenic Shock (LEVOECMO) trial investigated the use of levosimendan to facilitate ECMO weaning. However, levosimendan did not reduce the time to successful weaning and was associated with a higher incidence of ventricular arrhythmias (29). These findings suggest that inotropes, despite leading to an initial improvement in cardiac contractility, exacerbate cardiac injury through increased myocardial oxygen consumption, arrhythmogenicity, calcium overload, and proapoptotic signaling pathways (30–32).

Our findings underscore the need to critically reassess the need for inotropes after tMCS initiation, as organ perfusion becomes more dependent on device flow than on native cardiac output. This shift mitigates the initial indication for inotropes and may create a hemodynamic buffer that permits their safe de-escalation. Within this framework, recent studies have even explored the initiation of guideline-directed heart failure therapy under the “hemodynamic umbrella” of tMCS (33–36). Such approaches might be intuitively more feasible during micro-axial flow pump support as its intrinsic unloading effects reduce the risk for intracardiac thrombus formation and pulmonary edema. In the setting of VA ECMO support, adjunctive mechanical unloading strategies could potentially facilitate tapering of inotropic therapy, although robust evidence for the efficacy of intra-aortic balloon pump or Impella in this setting is still lacking and results of ongoing randomized clinical trials are eagerly awaited (37, 38).

### Strengths and Limitations

Our study is strengthened by the highly granular data structure, which provides a detailed description of different patient trajectories during VA ECMO support. Also, we applied forefront statistical methodologies which allowed us to assess the effects of cumulative inotropic exposure on outcomes and adjust for time-dependent confounding. The absence of standardized inotrope guidelines during VA ECMO likely led to significant practice variation, introducing a certain degree of randomness that strengthened the value of the observational data for our analyses (39). The robustness of our study findings was further underscored by several subgroup analyses which did not alter our findings. Nevertheless, due to the observational study design, residual confounding by unmeasured factors (e.g., LV distension and myocardial function) cannot be excluded, as inotropic therapy may function both as a modifiable exposure and as a marker of underlying disease severity (e.g., continuation due to lack of myocardial recovery). Our findings should therefore be considered hypothesis-generating and form a stepping stone for future studies aimed to infer causality. Additionally, as the study was conducted in Dutch centers only, the generalizability of our findings may be limited. Finally, although we explored potential differences in the association between β-adrenergic receptor agonists and PDEIs with ECMO weaning failure, an agent-specific analysis was not feasible due to frequent coadministration of inotropes and insufficient statistical power.

## CONCLUSIONS

In this observational study, a higher cumulative inotropic exposure during VA ECMO support was associated with an increased risk for ECMO weaning failure, 30-day mortality, and the occurrence of arrhythmias. Our findings suggest the need to critically reassess ongoing inotropic therapy after ECMO initiation, as default continuation might contribute to poorer outcomes. These results should be considered hypothesis-generating and require confirmation in future (interventional) studies that should systematically address the feasibility and safety of tapering or even stopping inotropic drugs in the setting of VA ECMO.

## ACKNOWLEDGMENTS

We thank Dr. M. Klimek, anesthesiologist at Erasmus MC, for his support as assessor at the Erasmus MC Graduate School.

## Supplementary Material
