# Supplementary material for: Time-Dependent Effects of Inotropic Exposure During Venoarterial Extracorporeal Membrane Oxygenation Support on the Probability of Extracorporeal Membrane Oxygenation Weaning Failure
Source: Crit Care Med. 2026 Sep 25;54(11):2911–23. doi: 10.1097/CCM.0000000000007338 (PMC13722429; doi:10.1097/CCM.0000000000007338)
Supplement: Supplementary file 1 [file ccm-54-2911-s001.pdf]

Time-dependent effects of inotrope exposure during V-A ECMO support on the probability of ECMO weaning failure

Myrthe P.J. van Steenwijk, Lara C.A. Pladet, Atreyu van Esch, Hong Jun Shi, Dirk W. Donker, Yannick Taverne, Jeroen J.H. Bunge, Carlos V. Elzo Kraemer, Jacinta J. Maas, Leon J. Montenij, Luuk Otterspoor, Erik Scholten, Laurien van Koppenhagen, Joost van Rosmalen, Diederik Gommers, Alina Constantinescu, Rudolf A. de Boer, Jacob. E. Møller, Dimitris Rizopoulos, Christiaan L. Meuwese

Online-Only Supplements

- Supplement 1. (Approval) Details of Participating centers
- Supplement 2. Prospectively defined definitions of relevant collected variables
- Supplement 3. Area under the curve of the Inotropic Score trajectory normalized per day
- Supplement 4. Joint model specification
- Supplement 5. Vasopressor score trajectories stratified by inotropic exposure groups
- Supplement 6. Number and percentage of missing values for each baseline and outcome variable
- Supplement 7. Rhythm at V-A ECMO initiation across tertiles of cumulative inotrope exposure during V-A ECMO support
- Supplement 8. Flowchart of study population selection
- Supplement 9. Changes in established hemodynamic parameters used for assessing adequate perfusion.
- Supplement 10. Temporal trajectories of hemodynamic parameters during the first 5 days of V-A ECMO support, stratified by inotrope exposure group.
- Supplement 11. Trajectory of time-varying confounders that are included in the joint model.
- Supplement 12. Sensitivity analysis per ECMO indication.
- Supplement 13. Illustration of competing event analysis
- Supplement 14. STROBE Statement—Checklist of items that should be included in reports of cohort studies

Supplement 1. (Approval) Details of Participating centers

**Hospital**	**Years of inclusion**	**IRB Board Name**	**Approval Number Local feasibility assessment**	**Date of local feasibility approval**
University Medical Center Utrecht (UMCU)	2016-2022	MEC-U	21/604	06-09-2021
Erasmus Medical Center Rotterdam (EMC)	2021-2022	MEC-U	MEC-2022-0067	21-03-2022
Leids Universitair Medisch Centrum (LUMC)	2017-2023	MEC-U	METc niet-wmo 21-604	06-05-2022
Antonius Ziekenhuis Nieuwegein	2021-2024	MEC-U	Z22.021	04-04-2022
Catharina Ziekenhuis Eindhoven	2022-2024	MEC-U	nWMO-2022.024	22-04-2022
Isala klinieken Zwolle	2021-2022	MEC-U	20220324	03-08-2022

Supplement 2. Prospectively defined definitions of relevant variables.

Variables		Definition
**Comorbidities**		
	Heart Failure	History of chronic heart failure with symptoms and signs, including patients with preserved, mid-range, and reduced ejection fraction. Diagnosis must be recorded prior to this current hospital-admission
	Myocardial infarction (MI)	History of definite myocardial infarction (EKG changes and/or enzyme changes) > 1 week before ICU admission. Diagnosis must be recorded prior to this current hospital-admission.
	Coronary artery disease (CAD)	History of coronary artery disease in patient file (obstruction of coronary arteries resulting in stable and/or unstable angina pectoris, STEMI, non-STEMI or retrospectively diagnosed myocardial infarction). Diagnosis must be recorded prior to this current hospital-admission.
	Coronary artery bypass graft (CABG)	History of coronary artery bypass grafting (CABG). Diagnosis must be recorded prior to this current hospital-admission.
	Percutaneous Coronary Intervention (PCI)	History of percutaneous coronary intervention (PCI). Diagnosis must be recorded prior to this current hospital-admission.
	Chronic Kidney Disease (CKD)	History of chronic kidney disease (mild – severe) reported in patient file. Diagnosis must be recorded prior to this current hospital-admission.
	Hypertension	History of hypertension (known with chronic > 1 month hypertension and/or antihypertensive medication). Diagnosis must be recorded prior to this current hospital-admission.
	Diabetes Mellitus (DM)	History of medication-dependent DM documented in patients file, including both uncomplicated or with end-organ damage. Diagnosis must be recorded prior to this current hospital-admission.
	Peripheral artery disease (PAD)	History of peripheral artery disease in patient file (intermittent claudication or past PTI/bypass for chronic arterial insufficiency, history of gangrene or acute arterial insufficiency, or untreated thoracic or abdominal aneurysm (≥6 cm) or dissection unless atherosclerosis has not been the reason for peripheral artery disease). Diagnosis must be recorded prior to this current hospital-admission.
	CVA/ TIA	History of transient ischemic attack (TIA) or cerebrovascular accident (CVA) or subarachnoidal haemorrhage (SAB) in patient file. Residual symptoms do not have to be present. Diagnosis must be recorded prior to this current hospital-admission.
	COPD	Chronic (over 6 months) usage of bronchodilator drugs or steroids indicated for chronic obstructive pulmonary disease. COPD must have been recorded prior to this current hospital-admission and the patient must be using medication indicated for COPD.

**Outcomes and Complications**		
	ECMO support duration	In days, including transition from V-A to veno-venous (V-V) ECMO support if applicable,
	The need for renal replacement therapy (RRT)	The initiation of RRT during ECMO support.
	Lactate clearance	Lactate clearance was defined as the time from V-A ECMO initiation until a patient's lactate reaches a level lower than 2 mmol/L. An important note in this definition is that delayed clearance may also reflect catecholamine-mediated metabolic effects, including β_3-adrenergic stimulation, rather than persistent tissue hypoxia alone.
	Occurrence of intracardiac or aortic root thrombosis	Presence of intracavitary cardiac thrombosis and/or aortic root thrombosis proven by echocardiography
	The need for mechanical LV unloading devices	Left ventricular overload as a results of V-A ECMO necessitating a left ventricular unloading technique: (one of the following)<br>· Intra- aortic balloon pump (IABP)<br>· Impella (micro-axial cardiac pump)<br>· Left ventricular vent<br>· Left atrial vent<br>· Iatrogenic atrial septal defect<br>· Pulmonary artery venting
	Arrhythmia occurrence	Defined as a documented (supra)ventricular tachycardia
	Vasopressor Score[1]	100 × norepinephrine (µg/kg/min) +<br>10,000 × vasopressin (U/kg/min) +<br>10 × Terlipressin (µg/min) +<br>10 × Phenylephrine (µg/min)
	Inotropic score (IS)[2] **=**	100 × epinephrine (µg/kg/min) +<br>10 × milrinone (µg/kg/min) +<br>dobutamine (µg/kg/min) +<br>dopamine (µg/kg/min) +<br>enoximone (µg/kg/min) +<br>50 × levosimendan (µg/kg/min)

Supplement 3. Area under the curve of the Inotropic Score normalized per day.

Example of three patients where cumulative inotropic exposure was determined. This was done by calculating the area under the curve (AUC) of the Inotropic Score (IS) trajectory per patient, which was then normalized by the number of ECMO support days, until a maximum of 7 days.

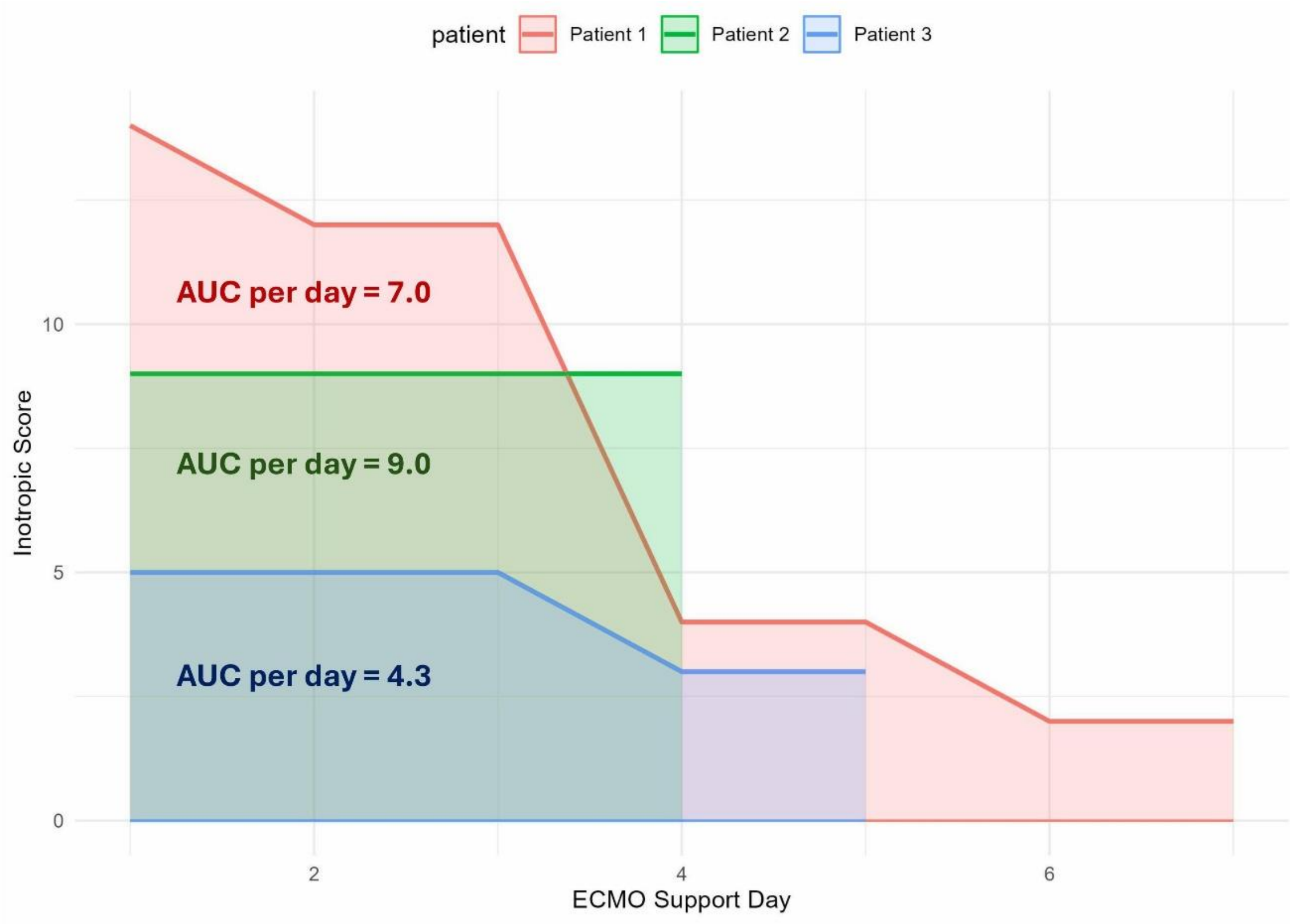

Supplement 4. Joint model specification

Linear Mixed Model for Inotropic Score:

The longitudinal trajectory of the inotropic score was modeled using a linear mixed-effects model (LMM). Fixed effects included age, Charlson Comorbidity Index (CCI), Acute Physiology and Chronic Health Evaluation (APACHE) IV score, V-A ECMO indication, cannulation configuration, and treating hospital. These variables were selected a priori based on their expected biological and clinical associations with inotropic dosing [15,16]. To account for within-subject correlation of repeated measurements, the model included both a random intercept and a random slope for time at the patient level.

The Cox proportional hazard submodel for ECMO weaning failure:

The time-to-event component was modeled using a Cox proportional hazards model, with ECMO weaning failure as the outcome. The model was adjusted for baseline age, sex, body mass index (BMI), CCI, APACHE IV score, vasopressor score prior to V-A ECMO initiation, indication for V-A ECMO, and the presence of a mechanical unloading device at baseline. Vasopressor score was calculated as a modification of the vasoactive–inotropic score (VIS), restricted to the vasopressor agents norepinephrine, vasopressin, and phenylephrine. These covariates were selected based on their potential confounding effects at ECMO initiation.

The proportional hazards assumption was assessed using scaled Schoenfeld residuals and was not violated.

Linear Mixed Model for time-varying confounders:

To account for potential time-dependent confounding, additional linear mixed-effects models were specified for the following longitudinal variables:

- Heart rate
- The occurrence of arrhythmias

- The placement of an LV unloading device during V-A ECMO support.

We specified each LMM with a random intercept and random slope for each patient to model the correlation between repeated measurements within single subjects.

Secondary outcomes

Time-to-event secondary outcomes included the occurrence of arrhythmias during ECMO-support, 30-day mortality, initiation of RRT, and lactate clearance. These secondary outcomes were also analyzed using separate joint models. Covariates were included in the fixed effect part of the model based on available literature about possible associations with exposing variables and outcomes for each association separately. Consistent with the primary model, all secondary outcome models also included a random intercept and slope to account for subject-level variability over time.

Supplement 5. Vasopressor score trajectories stratified by inotropic exposure groups

To assess potential statistical coupling between vasopressor scores and inotropic exposure groups over time, LOESS-smoothed trajectories with 95% confidence intervals were generated. At ECMO initiation, vasopressor scores were higher in the groups with greater inotropic exposure. However, subsequent vasopressor score trajectories were similar across exposure groups, with no significant correlation identified by Pearson analysis. In the joint model, vasopressor score was incorporated as a baseline covariate in the Cox proportional hazards submodel to adjust for differences in disease severity at baseline.

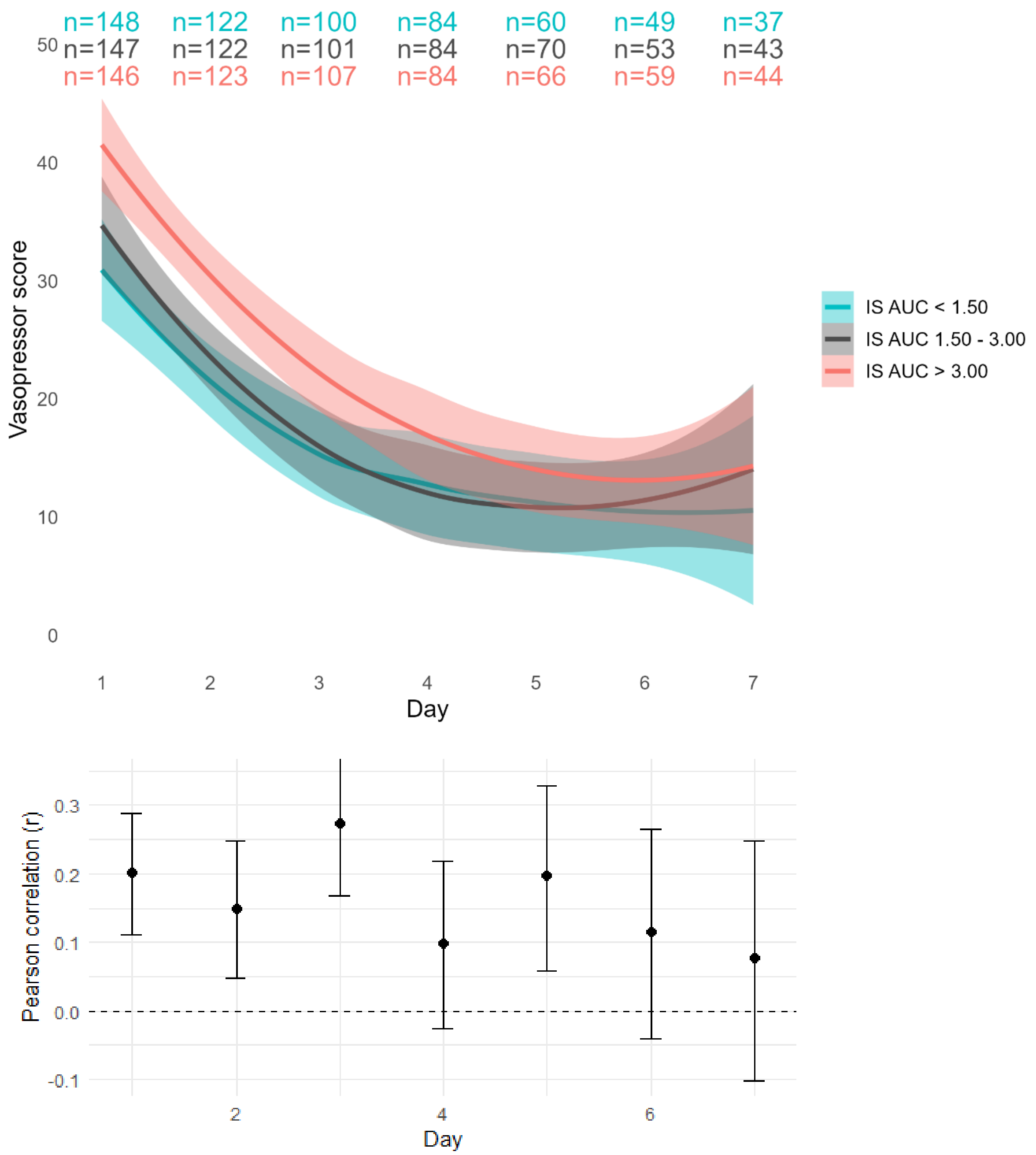

Supplement 6. Number and percentage of missing values for relevant variables

**Variable**	**Missing value, n (%)**
**Age, years**	**0 (0)**
**Sex**	**0 (0)**
**Body Mass Index**	**0 (0)**
**Medical history**	**0 (0)**
**Charlson comorbidity index**	**0 (0)**
**APACHE IV score**	**1 (0.2)**
**Vasopressor score prior to ECMO initiation**	**19 (4.3)**
**Heart rate at day 1**	**2 (0.5)**
**ECMO indication**	**0 (0)**
**Hospital**	**0 (0)**
**Unloading device at ECMO initiation**	**0 (0)**
**Rhythm at ECMO initiation**	**7 (1.6)**
**ICU length of stay**	**0 (0)**
**Hospital length of stay**	**2 (0.5)**
**Weaning failure**	**0 (0)**
**Mortality at 30 days**	**0 (0)**
**LVAD placement**	**0 (0)**
**The need for a heart transplantation**	**0 (0)**
**The initiation of renal replacement therapy**	**0 (0)**
**The occurrence of LV overload**	**0 (0)**
**The occurrence of arrhythmias**	**0 (0)**
**Lactate clearance**	**122 (27.7)**

Legend: ECMO = extracorporeal membrane oxygenation, V-A = veno-arterial, ICU = Intensive care unit, LVAD = left ventricular assist device

Supplement 7. Flowchart of study population selection.

This figure shows the patient selection based on in- and exclusion criteria of the study. This led to 440 eligible patients who were divided into three equal groups based on tertiles of the area under the curve (AUC) of the inotropic score (IS) normalized by the number of ECMO support days, which ranged from 0 to 52.0 (median 2.5, IQR: 1.0 – 5.0). Based on tertiles of this AUC IS per day, low, moderate, and high IS exposure groups were defined as <1.50, 1.50–3.00, and >3.00, respectively.

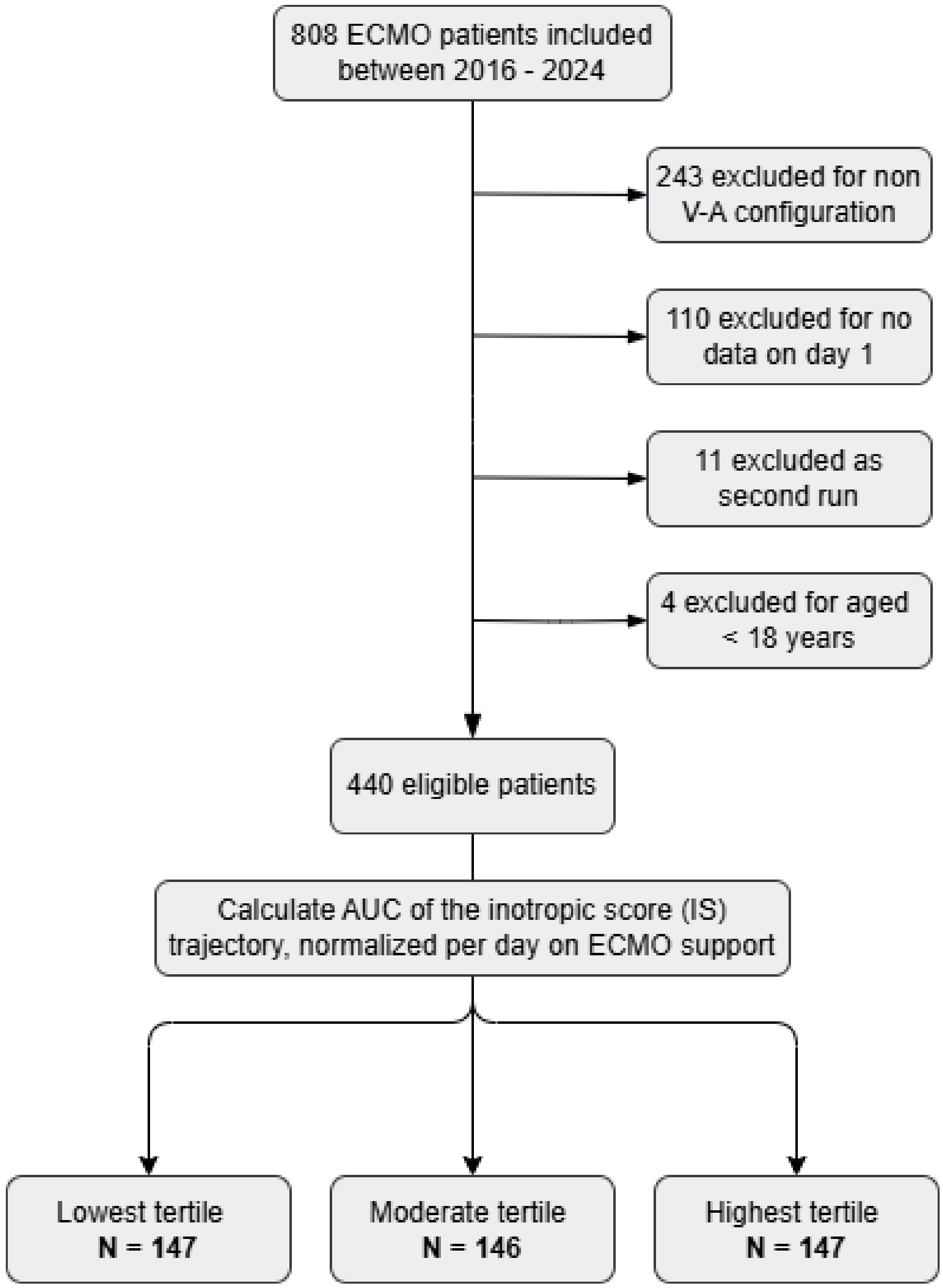

Legend: ECMO = extracorporeal membrane oxygenation, V-A = veno-arterial, AUC = area under the curve

Supplement 8. Rhythm at V-A ECMO initiation across tertiles of cumulative inotrope exposure during V-A ECMO support

	Characteristic	Total	Lowest tertile<br>(IS AUC < 1.50)	Moderate tertile<br>(IS AUC 1.50 - 3.00)	Highest tertile<br>(IS AUC > 3.00)	p-value
**Number of patients**		**440**	**147**	**147**	**146**	
**Inotropic drugs (%)**						
	Any inotropic drug	338 (76.8)	53 (35.8)	141 (95.9)	144 (98.0)	0.551
	Epinephrine	32 (7.2)	1 (0.7)	5 (3.4)	26 (17.7)	0.573
	Dobutamine	162 (36.7)	15 (10.1)	48 (32.7)	99 (67.3)	0.022
	Dopamine	6 (1.4)	1 (0.7)	2 (1.4)	3 (2.0)	0.030
	Milrinone	147 (33.3)	35 (23.6)	60 (40.8)	52 (35.4)	0.592
	Enoximone	89 (20.1)	5 (3.4)	46 (31.3)	38 (25.9)	0.088
	Levosimendan	1 (0.2)	0 (0.0)	1 (0.7)	0 (0.0)	0.281
**Hospital (%)**						
	Academic	352 (80.0)	115 (78.2)	111 (75.5)	126 (86.3)	0.053
**Rhythm (%) ***						0.129
	Sinus rhythm	323 (73.1)	110 (74.3)	114 (77.6)	99 (67.3)	
	Paced rhythm	66 (14.9)	22 (14.9)	15 (10.2)	29 (19.7)	
	SVT	37 (8.4)	10 (6.8)	15 (10.2)	12 (8.2)	
	VT	3 (0.7)	1 (0.7)	0 (0.0)	2 (1.4)	

	Other	11 (2.9)	4 (3.4)	3 (2.0)	4 (3.4)	

IS = inotropic score, AUC = area under the curve, ECMO = ExtraCorporeal Membrane Oxygenation, SVT = supraventricular tachycardia, VT = ventricular tachycardia

* Categorical variables were compared using a Chi-Square test or Fisher exact test

Supplement 9. Changes in established hemodynamic parameters used for assessing adequate perfusion.

The panel includes the trajectory of (A) Mean Arterial Pressure (MAP), (B) Lactate levels, (C) Cardiac output as measured by pulmonary artery catheterization, and (D) Central or mixed venous oxygen. Saturation, stratified by tertiles based on the Inotropic Score (IS) normalized by the number of ECMO support days. Patients with higher mean IS values, exhibited lower MAPs, cardiac output and venous oxygen saturation values, with higher lactate levels prior to V-A ECMO initiation. Apart from cardiac output measurement, performed in a very limited number of patients, the rest of the hemodynamic parameters tended to converge during V-A ECMO support.

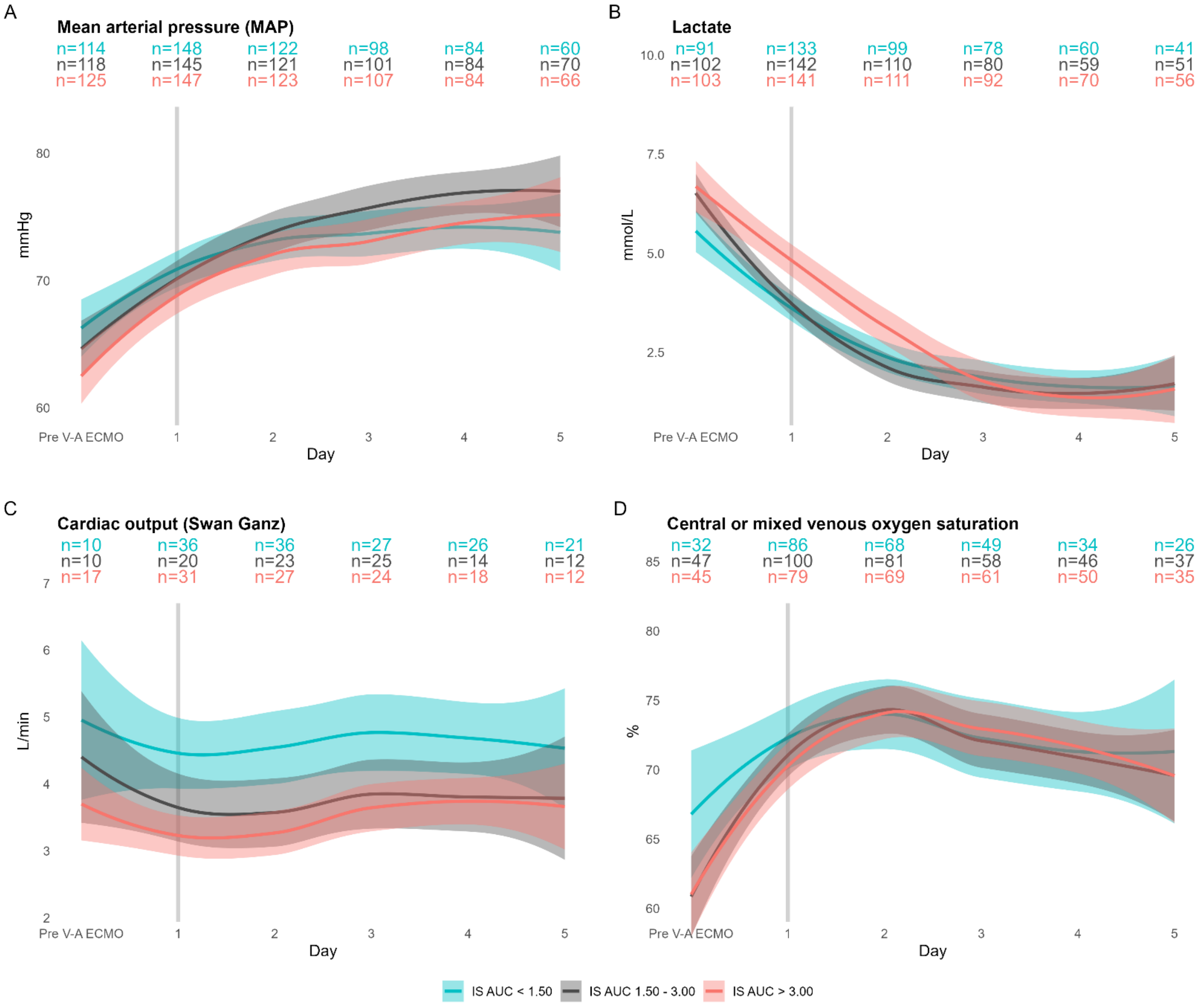

Supplement 10. Temporal trajectories of hemodynamic parameters during the first 5 days of V-A ECMO support, stratified by inotrope exposure group.

The eight panels display individual data points with LOESS-smoothed trajectories and corresponding 95% confidence interval for (A) Heart rate (HR), (B) Mean arterial pressure (MAP), (C) Sequential Organ Failure Assessment (SOFA), (D) Serum lactate concentration, (E) Daily fluid balance (mL) , (F) FiO_2 requirement, (G) V-A ECMO flow (L/min), and (H) V-A ECMO sweep gas flow (L/min), during the first 5 days of V-A ECMO support, stratified by inotropic score exposure group. Patient numbers contributing to each time point are shown.

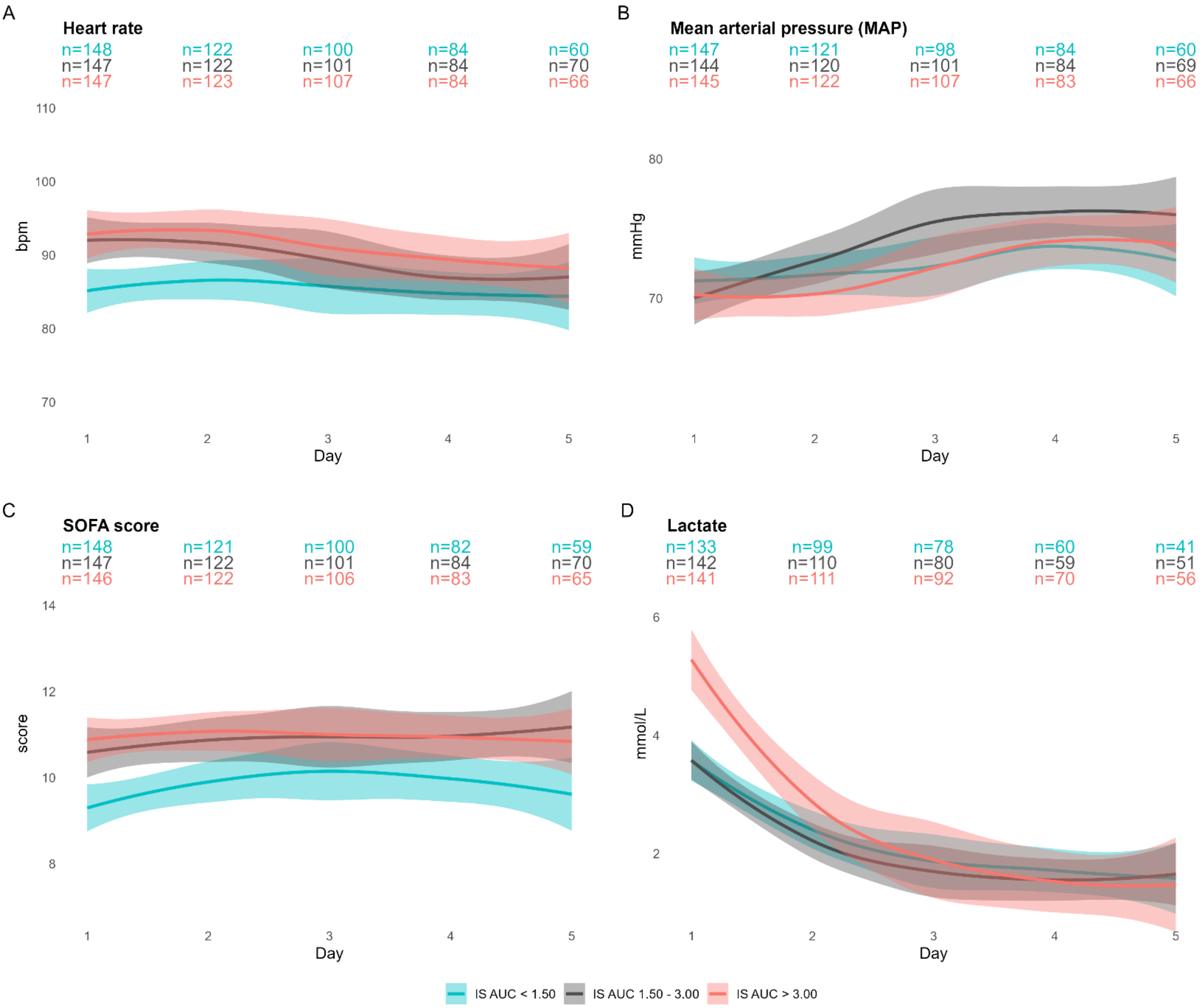

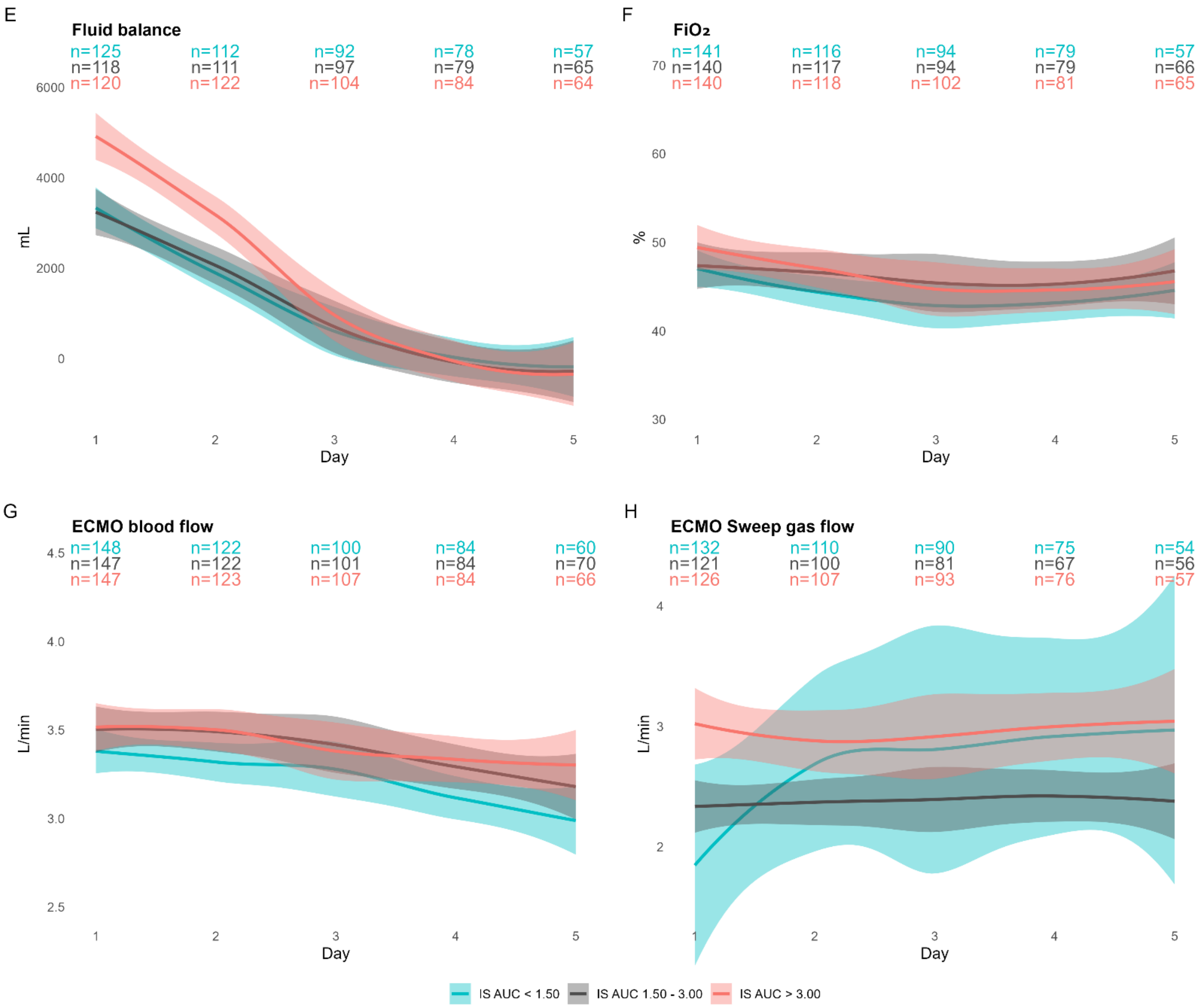

E
Fluid balance
n=125 n=112 n=92 n=78 n=57
n=118 n=111 n=97 n=79 n=65
n=120 n=122 n=104 n=84 n=64
mL
Day
F
FiO₂
n=141 n=116 n=94 n=79 n=57
n=140 n=117 n=94 n=79 n=66
n=140 n=118 n=102 n=81 n=65
%
Day
G
ECMO blood flow
n=148 n=122 n=100 n=84 n=60
n=147 n=122 n=101 n=84 n=70
n=147 n=123 n=107 n=84 n=66
L/min
Day
H
ECMO Sweep gas flow
n=132 n=110 n=90 n=75 n=54
n=121 n=100 n=81 n=67 n=56
n=126 n=107 n=93 n=76 n=57
L/min
Day
IS AUC < 1.50
IS AUC 1.50 - 3.00
IS AUC > 3.00

Supplement 11. Trajectory of time-varying confounders that are included in the joint model.

A) Trajectory of the mean heart rate with its corresponding 95% confidence interval during V-A ECMO support. *B*) Bar graph showing the proportion of patients who experienced arrhythmias during ECMO support. Points indicate the mean percentage of patients with arrhythmias for each day, and vertical lines represent the corresponding 95% confidence intervals. *C*) Similarly as for arrhythmias, the proportion of patients experiencing left ventricular (LV) overload during V-A ECMO support is presented using bar graphs. LV overload is defined as the need for a interventional LV unloading technique during V- A ECMO support (e.g. Impella, intra-aortic balloon pump)

A)

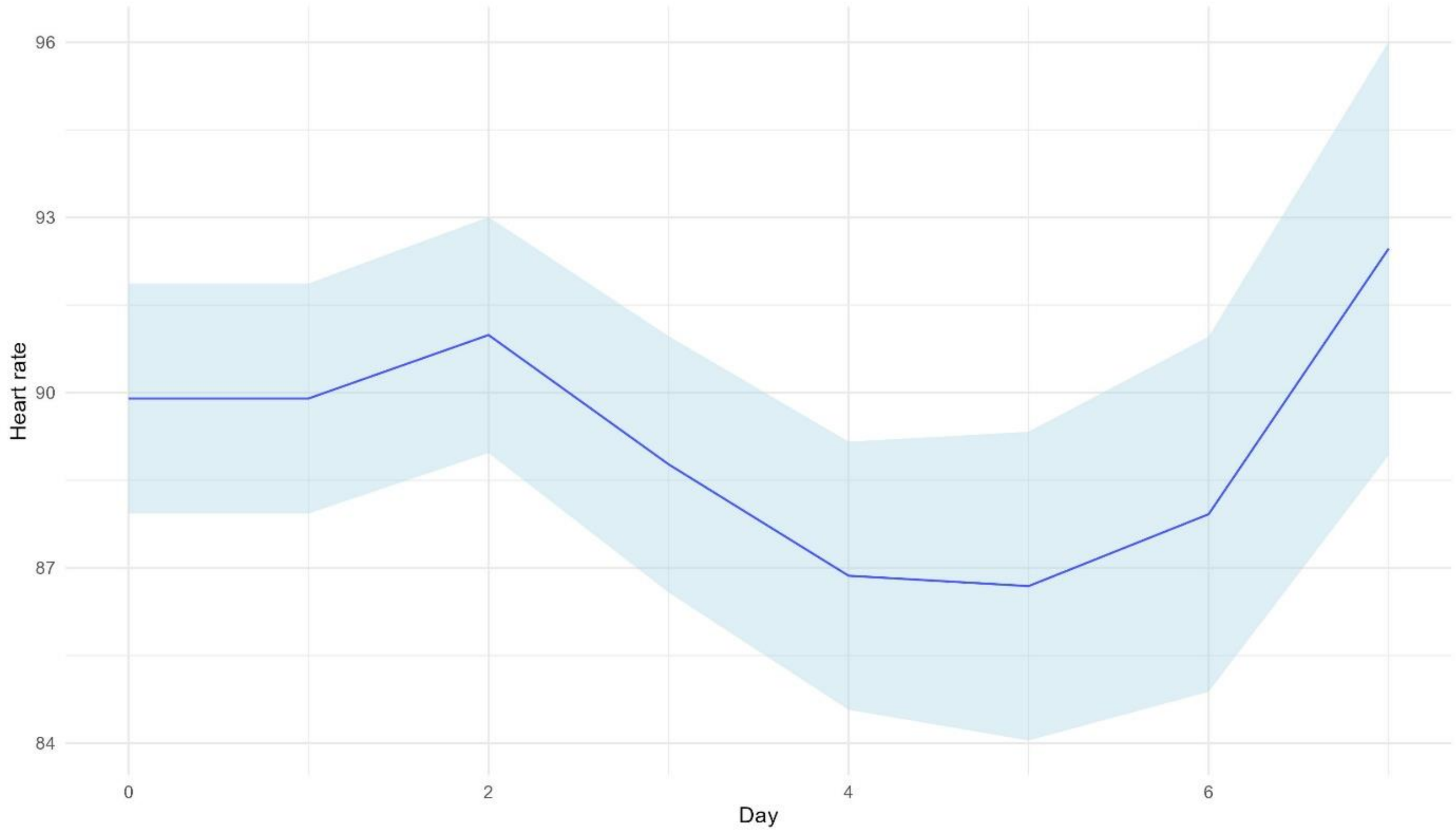

B)

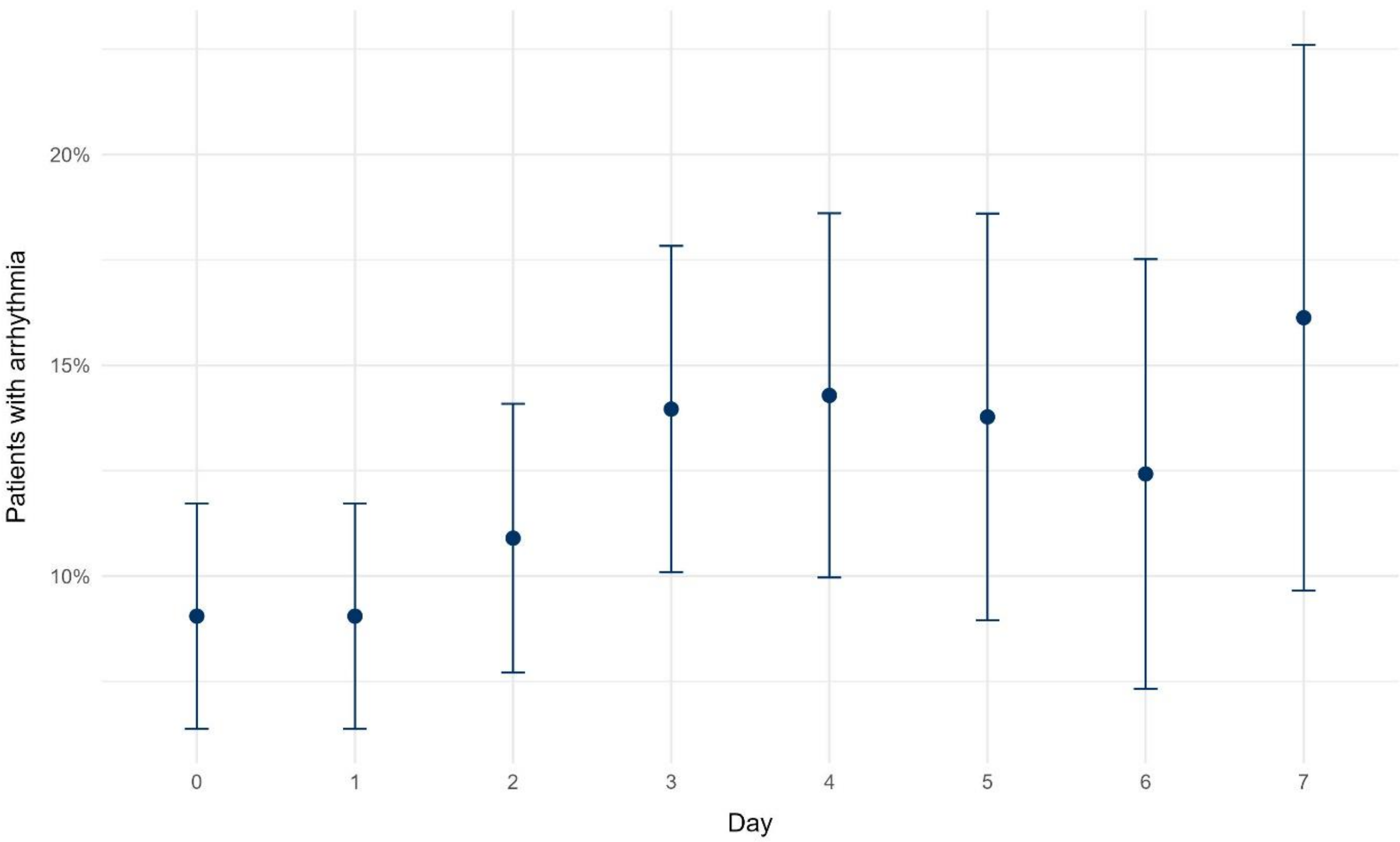

20%
15%
10%
Patients with arrhythmia
0
1
2
3
4
5
6
7
Day

C)

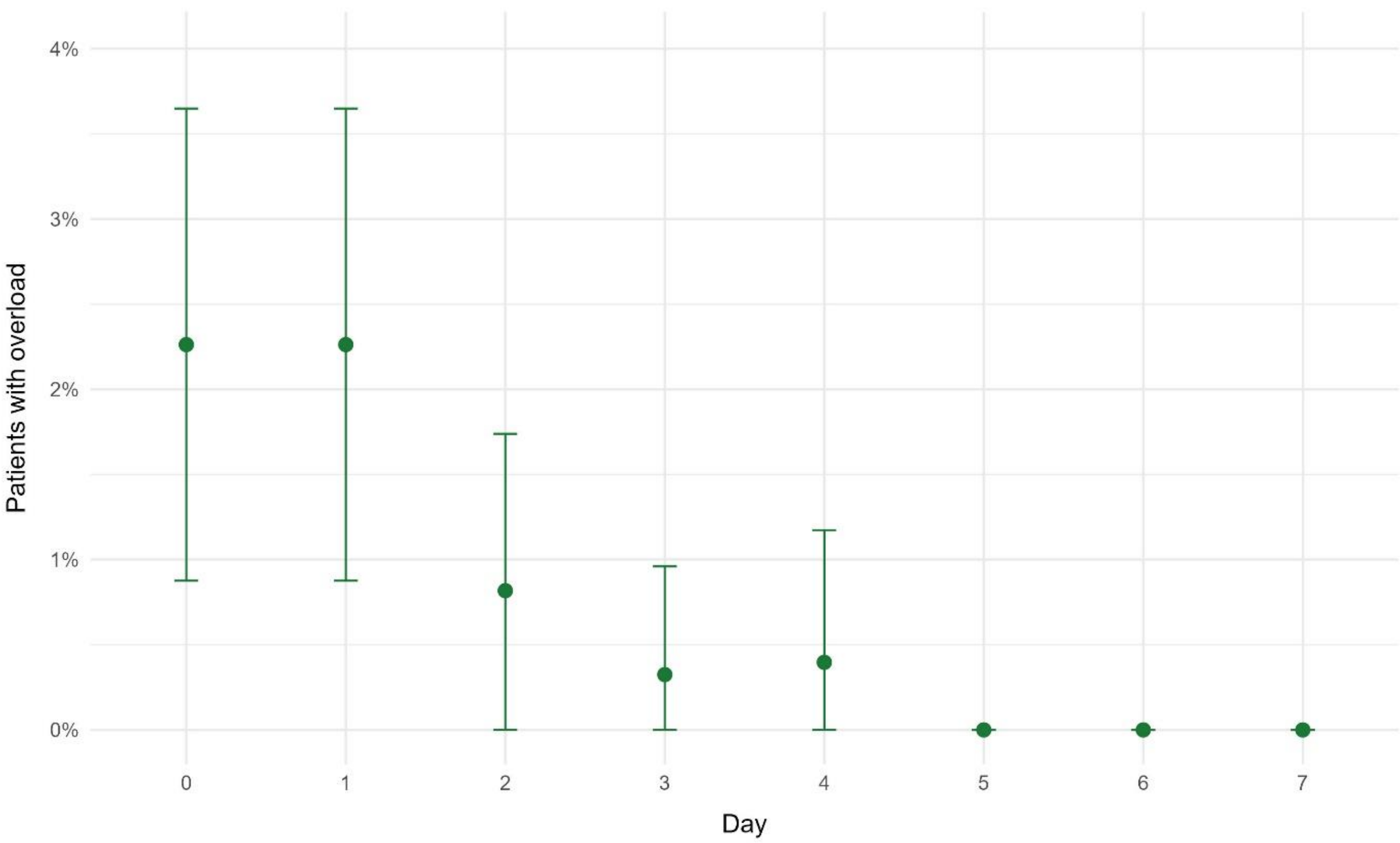

4%
3%
2%
1%
0%
Patients with overload
0
1
2
3
4
5
6
7
Day

Supplement 12. Sensitivity analysis per ECMO indication.

This figure shows the outcomes of Cox regression analyses on the effect of cumulative inotrope exposure on ECMO weaning failure in i) a subgroup where ECPR patients were excluded (n = 327), and ii) solely post-cardiotomy patients (n =164). A Cox regression model was used because a joint model was a too complex to fit in these subgroups. Outcomes are presented as hazard ratio with their 95% confidence interval.

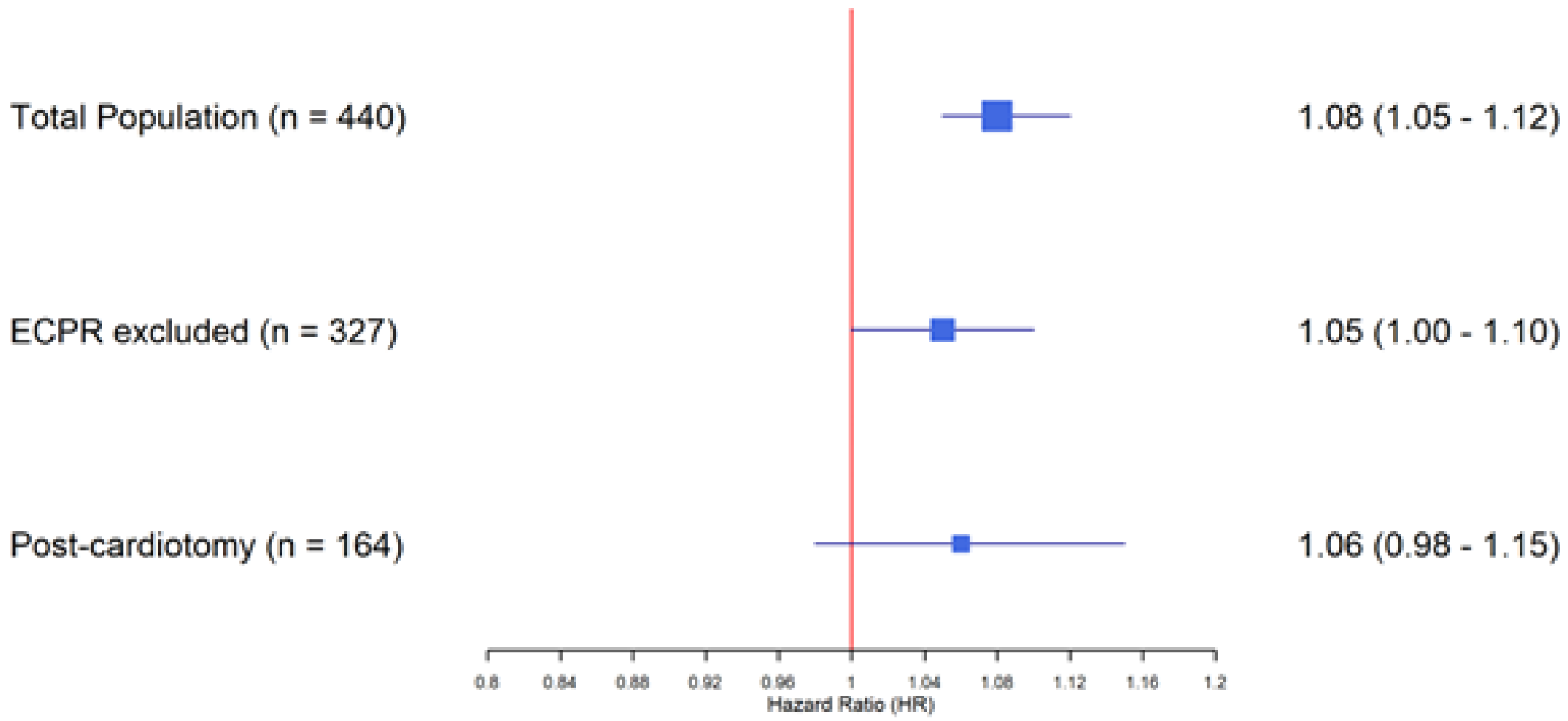

Supplement 13. Competing event analysis

In this figure the outcome of the competing risk analysis until 30 days after ECMO initiation is showed. This analysis accounts for neurological death as competing risk as this precludes the observation of ECMO weaning due to poor cardiac recovery. The blue curve represents ECMO weaning failure over time, the green curve shows the patients that were not successfully weaned from ECMO due to neurological causes, and red indicates the patients that were successfully weaned from ECMO at 30 days after initiation, which is shown as a single step at day 30, reflecting the defined censoring time.

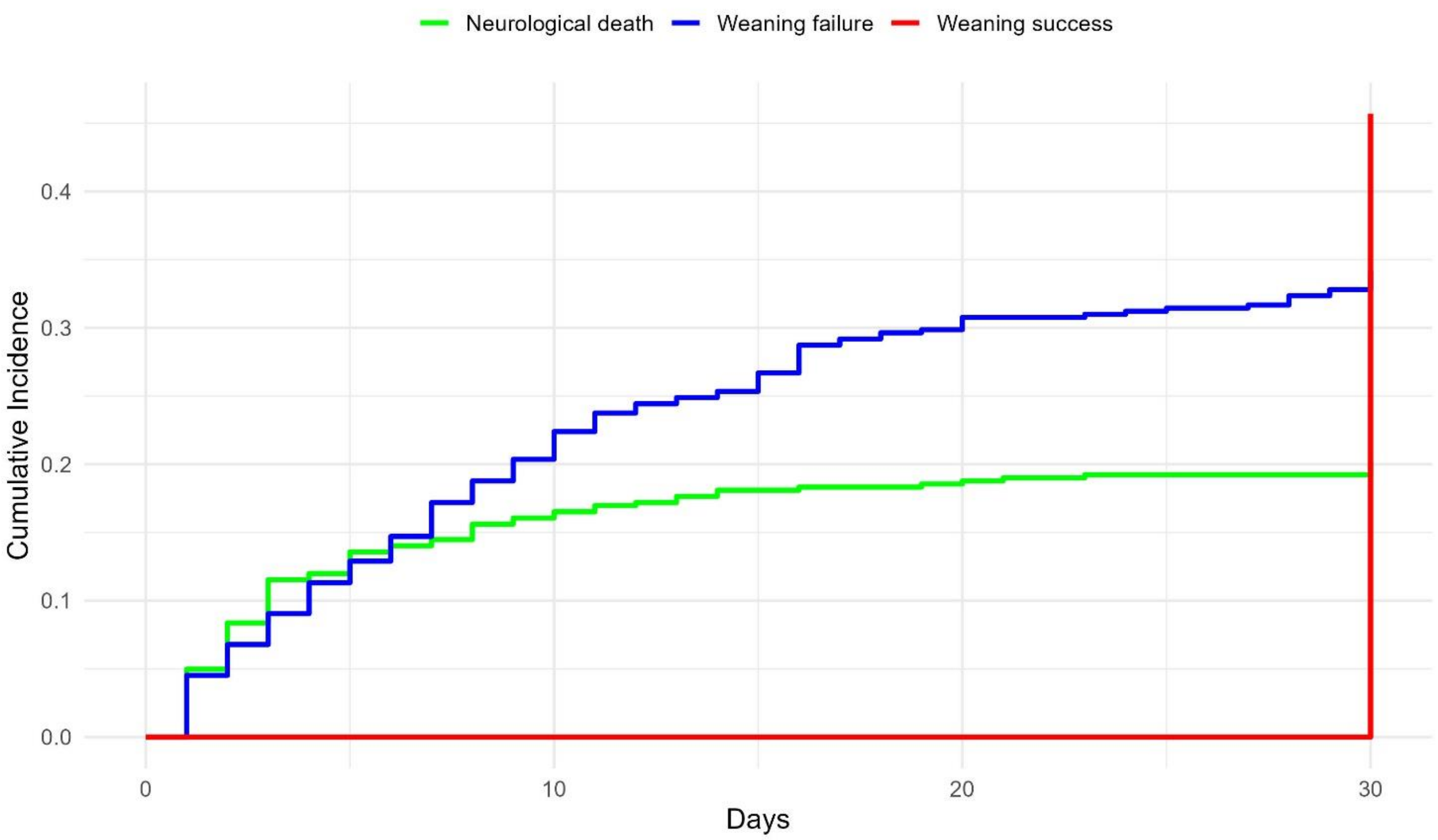

Supplement 14. STROBE Statement—Checklist of items that should be included in reports of cohort studies

	Item No	Recommendation	
**Title and abstract**	1	(*a*) Indicate the study's design with a commonly used term in the title or the abstract	Page 4
		(*b*) Provide in the abstract an informative and balanced summary of what was done and what was found	Page 4
**Introduction**			
Background/rationale	2	Explain the scientific background and rationale for the investigation being reported	Page 6
Objectives	3	State specific objectives, including any prespecified hypotheses	Page 6
**Methods**			
Study design	4	Present key elements of study design early in the paper	Page 7
Setting	5	Describe the setting, locations, and relevant dates, including periods of recruitment, exposure, follow-up, and data collection	Page 7
Participants	6	(*a*) Give the eligibility criteria, and the sources and methods of selection of participants. Describe methods of follow-up	Page 7
		(*b*) For matched studies, give matching criteria and number of exposed and unexposed	N.A.
Variables	7	Clearly define all outcomes, exposures, predictors, potential confounders, and effect modifiers. Give diagnostic criteria, if applicable	Page 8
Data sources/ measurement	8*	For each variable of interest, give sources of data and details of methods of assessment (measurement). Describe comparability of assessment methods if there is more than one group	Page 7 – 8
Bias	9	Describe any efforts to address potential sources of bias	Page 9 - 10
Study size	10	Explain how the study size was arrived at	Page 11
Quantitative variables	11	Explain how quantitative variables were handled in the analyses. If applicable, describe which groupings were chosen and why	Page 8 - 9
Statistical methods	12	(*a*) Describe all statistical methods, including those used to control for confounding	Page 8 - 10
		(*b*) Describe any methods used to examine subgroups and interactions	Page 9 - 10
		(*c*) Explain how missing data were addressed	Page 8
		(*d*) If applicable, explain how loss to follow-up was addressed	N.A.
		(*e*) Describe any sensitivity analyses	Page 10
**Results**			
Participants	13*	(a) Report numbers of individuals at each stage of study—eg numbers potentially eligible, examined for eligibility, confirmed eligible, included in the study, completing follow-up, and analysed	Page 11
		(b) Give reasons for non-participation at each stage	Page 11

		(c) Consider use of a flow diagram	Supplement 4
Descriptive data	14*	(a) Give characteristics of study participants (eg demographic, clinical, social) and information on exposures and potential confounders	Page 10, table 1
		(b) Indicate number of participants with missing data for each variable of interest	Supplement 3
		(c) Summarise follow-up time (eg, average and total amount)	N.A.
Outcome data	15*	Report numbers of outcome events or summary measures over time	Page 11 – 12 , table 2 -3
Main results	16	(*a*) Give unadjusted estimates and, if applicable, confounder-adjusted estimates and their precision (eg, 95% confidence interval). Make clear which confounders were adjusted for and why they were included	Page 11 – 12 and Table 2
		(*b*) Report category boundaries when continuous variables were categorized	Page 11
		(*c*) If relevant, consider translating estimates of relative risk into absolute risk for a meaningful time period	N.A.
Other analyses	17	Report other analyses done—eg analyses of subgroups and interactions, and sensitivity analyses	Page 12 + Supplement
**Discussion**			
Key results	18	Summarise key results with reference to study objectives	Page 13
Limitations	19	Discuss limitations of the study, taking into account sources of potential bias or imprecision. Discuss both direction and magnitude of any potential bias	Page 14
Interpretation	20	Give a cautious overall interpretation of results considering objectives, limitations, multiplicity of analyses, results from similar studies, and other relevant evidence	Page 14
Generalisability	21	Discuss the generalisability (external validity) of the study results	Page 14
**Other information**			
Funding	22	Give the source of funding and the role of the funders for the present study and, if applicable, for the original study on which the present article is based	Page 3

References

1. Overgaard CB, Dzavík V. Inotropes and vasopressors: review of physiology and clinical use in cardiovascular disease. Circulation. 2008;118(10):1047-56.
2. Bangalore H, Gaies M, Ocampo EC, Heinle JS, Guffey D, Minard CG, et al. The Total Inotrope Exposure Score: an extension of the Vasoactive Inotrope Score as a predictor of adverse outcomes after paediatric cardiac surgery. Cardiol Young 2017;27(6):1146 - 52.
